# Supplementary material for: A network of coiled-coil and actin-like proteins controls the cellular organization of magnetosome organelles in deep-branching magnetotactic bacteria
Source: Nat Commun. 2025 Dec 11;16:11453. doi: 10.1038/s41467-025-66326-2 (PMC12748827; doi:10.1038/s41467-025-66326-2)
Supplement: Supplementary file 1 — Supplmentary Information [file 41467_2025_66326_MOESM1_ESM.pdf]

Supplementary Notes:

Protein abundance during hydrogen-induced inhibition of magnetosome synthesis

To investigate whether hydrogen affects magnetosome protein expression we compared the proteomes of RS-1 cells grown under hydrogen or nitrogen using liquid chromatography-mass spectrometry. Most magnetosome gene cluster (MGC) proteins showed similar levels, except Mad10, which was over 20 times more abundant in nitrogen (Supplemental Figure 11 A and B). However, Mad10 is not responsible for magnetosome inhibition under hydrogen conditions, as indicated by its magnetosome formation phenotype. In hydrogen-grown cells, hydrogenases, a response regulator, a signaling protein, and the ferric uptake regulator, Fur, were more abundant, while nitrogen-grown cells had higher levels of dehydrogenases and a carbon storage regulator (Supplemental Figure 11 C-F). These findings suggest potential molecular mechanisms for hydrogen's effect on magnetosome formation, warranting further investigation in the future.

Architecture of Chain organization proteins in RS-1

For each chain organization gene deleted in RS-1, predicted protein structures were modeled to visualize their structural features. Mad20, Mad25, and Mad26 all exhibited similar protein structures, characterized by long coiled-coil filament-like regions (Supplemental Figure 8 C-E). Similar features are seen in other bacterial species such as the coiled-coil protein, Crescentin, found in *Caulobacter crescentus*[1]. These coiled-coil domains are predicted to facilitate strong protein-protein interactions, which was supported by bacterial two-hybrid assays, where all three proteins interacted extensively with each other as well as with other chain organization proteins (Figure 6). Mad10 and Mad23, in contrast, have much shorter coiled-coil regions (Supplemental Figure 1 A), which is also reflected in their 3D structural models (Supplemental Figure 8 A-B). While Mad10 lacks additional predicted domains, previous studies suggest it contains a magnetite-binding region[2]. Mad23, however, possesses a distinctive HEAT repeat domain in addition to its small coiled-coil region (Supplemental Figure 1 A and Supplemental Figure 8 B-ii). HEAT domains are typically associated with protein-protein interactions, a hypothesis further supported by bacterial two-hybrid assays, where Mad23 showed strong interactions with Mad10 and Mad25 (Figure 6).

Both Mad28 and MamK are predicted to contain actin-like domains and share highly similar predicted protein structures (Supplemental Figure 1 A and Supplemental Figure 8 F-G). To further investigate their relationship, we performed both 3D structural and protein sequence alignments. While the sequence alignment revealed low similarity (Supplemental Figure 8 J),

the 3D structural alignment showed strong alignment between the two proteins (Supplemental Figure 8 H), suggesting that despite sequence divergence, their structural similarities may indicate conserved functions. However, our genetic studies and biomineralization time course analyses revealed distinct roles: MamK is essential for subchain formation, while Mad28 is required for proper chain localization along the positive curvature (Figure 5F-G). Additionally, the original *mad28* sequence from RS-1 in the NCBI database before 2019 lacked the first 114 nucleotides or 38 amino acids, shown in green in Supplemental Figure 8 H-J. However, this region is essential for restoring the wild-type phenotype in *mad28* complementation experiments (Supplemental Figure 6 F). Notably, this sequence is unique to Mad28 and does not align with MamK in the 3D structural comparison (Supplemental Figure 8 H). This specific *mad28* region is also present in Mad28 homologs from other deep-branching MTB, further supporting its functional significance. Supplemental Figure 8-I highlights this region in green in MYR-1, a deep-branching MTB from the *Nitrospirota* phylum.

Supplementary Figures:

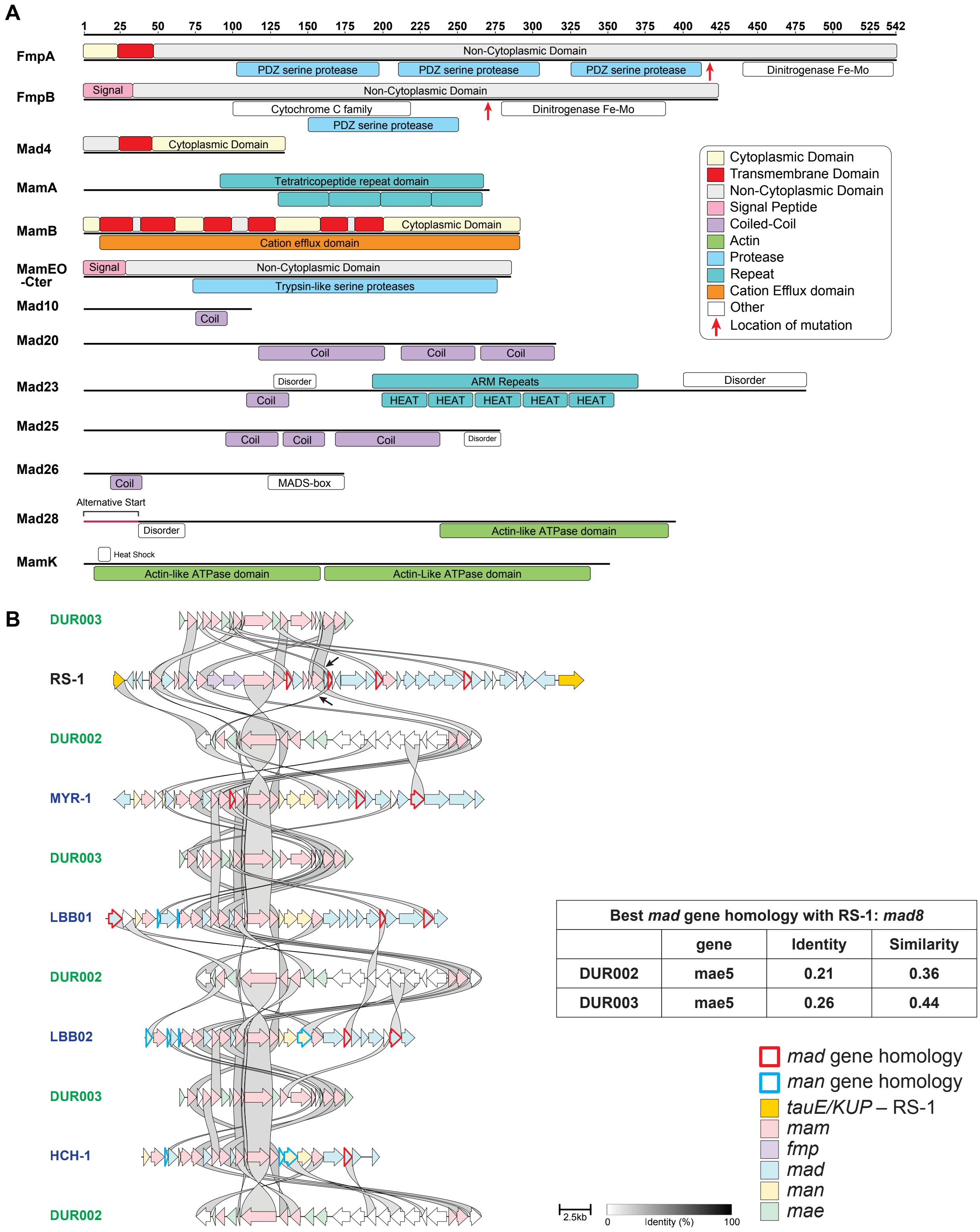

Best *mad* gene homology with RS-1: *mad8*			
	**gene**	**Identity**	**Similarity**
**DUR002**	**mae5**	**0.21**	**0.36**
**DUR003**	**mae5**	**0.26**	**0.44**

Supplemental Figure 1. *Protein domains of magnetosome genes in this study and mad gene comparisons between deep branching MTB.*

(A) Domains of proteins studied here. Protein domains were identified using InterPro [3] and uploaded on to Geneious [4] to generate domain maps. Transmembrane domains were additionally verified using DeepTMHMM[5]. Red arrows on FmpA and FmpB indicate the location of their mutations for $fmpA^{Q418*}$ and $fmpB^{808delG}$ mutants. Mad28 shows an alternative start site that was not included in the original NCBI annotation. Importantly, Δ*mad28* mutant could only be complemented when the alternative start site was included. (B) Magnetosome gene cluster comparison between DUR002 and DUR003 *(Elusimicrobiota)* and RS-1 *(Desulfobacterota* representative*),* and between DUR002 and DUR003 and MYR-1, LBB01, LBB02, and HCH-1 *(Nitrospirota* representatives*)*. It has been stated that DUR002 and DUR003 do not contain any mad genes[6]. However, this analysis indicates that they do have homologues to some *mad* genes. MGCs were extracted from genomes available on NCBI manually and compared with Clinker[7]. Genes outlined in red are *mad* genes that show some homology in either DUR002 or DUR003. Genes outlined in blue are *man* genes that show some homology in either DUR002 or DUR003. The table on the right shows the proportions of identity and similarity between *mad8* of RS-1 and its homolog, *mae5*, which is found in both DUR002 and DUR003. The black arrows indicate the *mad8* homology on the comparison map. *mad4*, *mad11* and *mad24* from RS-1 also have homologs, but only in DUR003. In addition, *mad24* from HCH-1 and LBB02 has a homolog to a gene in the MGC of DUR003.

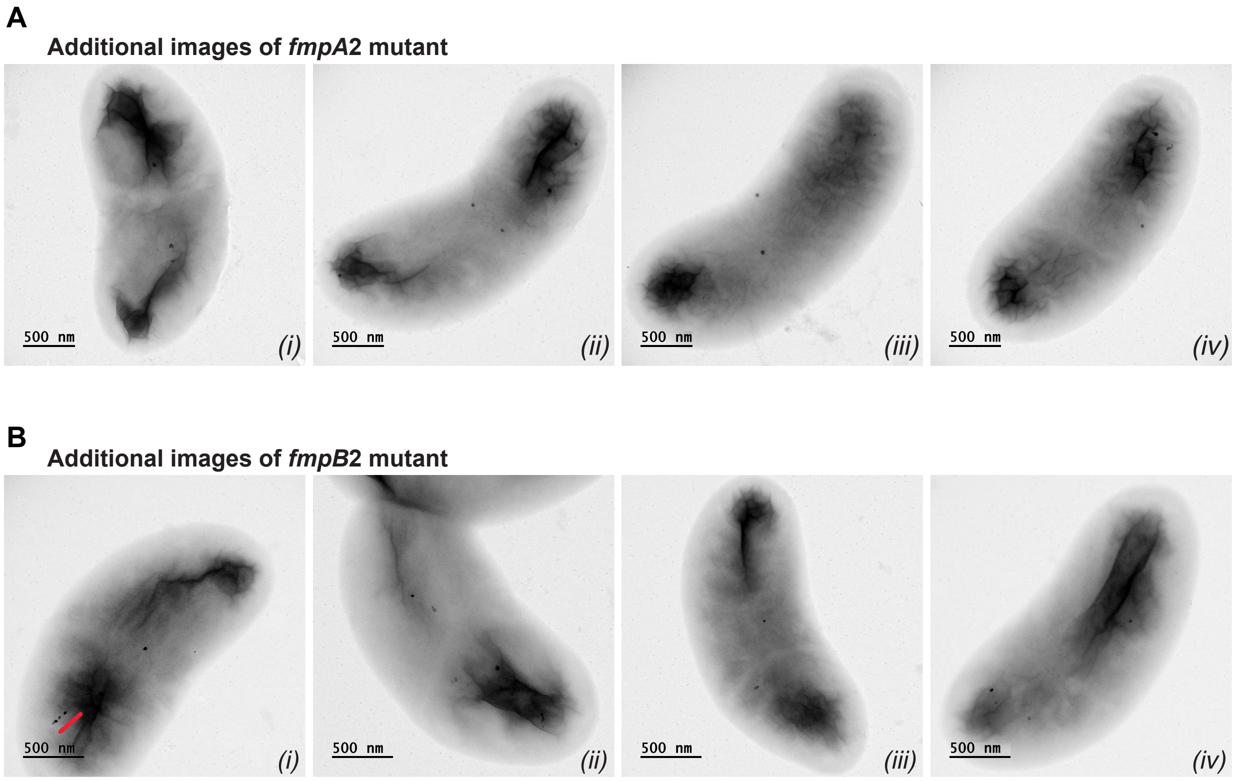

Supplemental Figure 2. *Additional TEM images of fmpA and fmpB mutants.*

(A) Additional TEM images of *fmpA* mutant in RS-1. These images illustrate fewer and misshaped crystals in each cell to complement the statistical data from Figure 4 and Supplemental Figure 7. (B) Additional TEM images of *fmpB* mutant in RS-1. These images illustrate the fewer and smaller crystals in each cell to complement the statistical data from Figure 4 and Supplemental Figure 7. Additionally, B-*i* shows an example of a chain phenotype in this mutant.

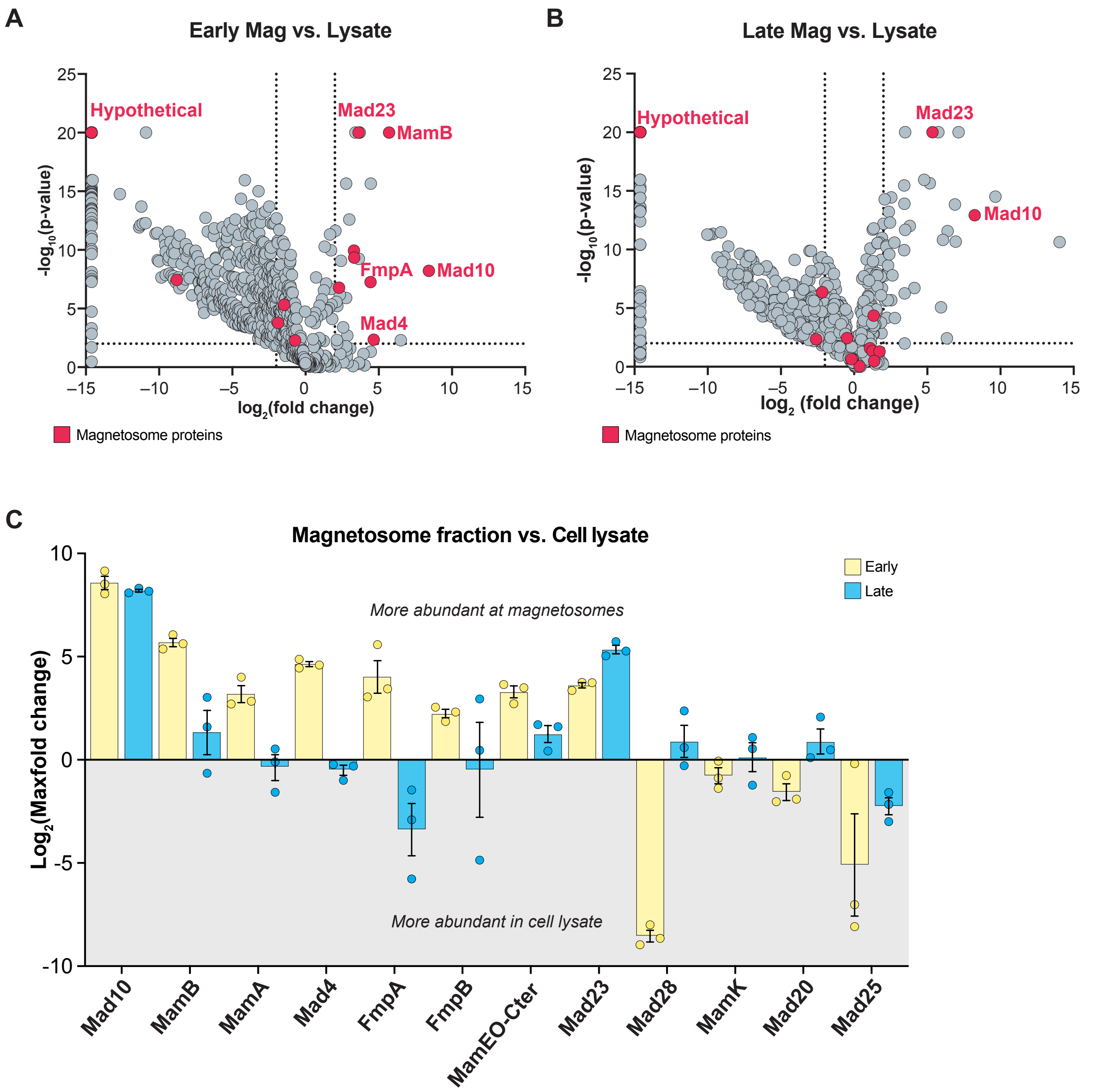

Supplemental Figure 3. *Additional proteomics data for early vs. late stage of biomineralization.* (A-B) A volcano plots showing the differential abundance analysis between magnetosome fraction and whole cell lysate for the early (A) and late (B) stages of biomineralization. Red dots indicate proteins from the magnetosome gene cluster (DMR_040710 – DMR_41530). Proteins with a peptide abundance of 0 in the magnetosome fraction result in an infinite fold change. Therefore, their fold change was arbitrarily set to 25,000. This is reflected in the volcano plots as a $\log_2$ value of -15. Additionally, any p-value of 0 was arbitrarily set to a $-\log_{10}$(p-value) of 20. (C) Abundance of magnetosomes proteins (those encoded by MGC genes) in the cell lysate versus the magnetosome fraction for three biological replicates. The abundance of magnetosome

proteins is represented as greater than 0 when they are more prevalent in the magnetosome fraction and as less than 0 when they are more abundant in the cell lysate. The error bars represent +/- SEM. Yellow bars represent early stage proteins abundances and blue bars represent late stage proteins abundances.

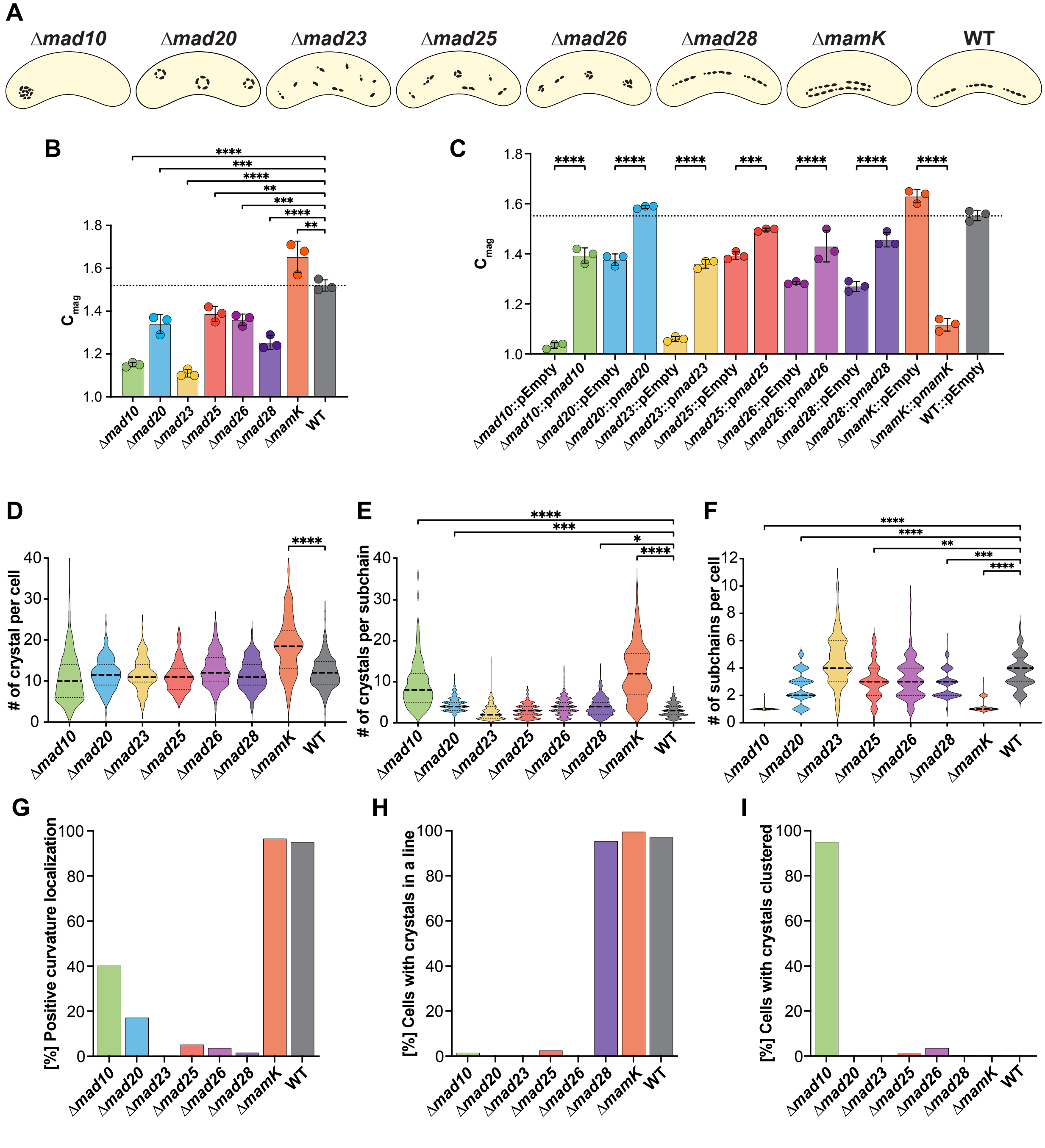

Supplemental Figure 4. *Characterization of magnetosome gene deletions in RS-1*

(A) Cartoon diagram of representative phenotype of each strain. (B) C_{mag} values are shown for each strain without plasmids. Error bars represent the standard deviation from the mean of three biological replicates. One-way ANOVA comparing C_{mag} values showed significant differences for all mutants compared to WT: Δ*mad10* ($p < 0.0001$), Δ*mad20* ($p = 0.0002$), Δ*mad23* ($p < 0.0001$), Δ*mad25* ($p = 0.0030$), Δ*mad26* ($p = 0.0005$), Δ*mad28* ($p < 0.0001$), and Δ*mamK* ($p = 0.0030$) (Supplemental Table 9). (C) C_{mag} values are shown for each mutant carrying either an empty plasmid or a plasmid constitutively expressing the deleted gene. The WT strain carries only an empty plasmid. Error bars indicate the standard deviation from the mean of three biological replicates. One-way ANOVA comparing mutants with empty plasmids to their corresponding complemented strains revealed significant differences for all mutants: Δ*mad10*::pMad10 ($p < 0.0001$), Δ*mad20*::pMad20 ($p < 0.0001$), Δ*mad23*::pMad23 ($p < 0.0001$), Δ*mad25*::pMad25 ($p = 0.0002$), Δ*mad26*::pMad26 ($p < 0.0001$), Δ*mad28*::pMad28 ($p < 0.0001$), and Δ*mamK*::pMamK ($p < 0.0001$), compared to their respective mutants carrying empty plasmids (Supplemental Table 9). The dotted line in panels (B) and (C) indicates the average C_{mag} of WT. (D) Violin plots displaying the number of crystals per cell for each strain. (E) Violin plots illustrating the number of crystals per subchain for each strain. (F) Violin plots representing the number of subchains per cell for each strain. (D) One-way ANOVA comparing all mutants to WT showed a significant difference in magnetosomes per cell only for ΔmamK ($p < 0.0001$; Supplemental Table 9). (E) One-way ANOVA comparing magnetosomes per subchain revealed significant differences for Δ*mad10* ($p < 0.0001$), Δ*mad20* ($p = 0.0001$), Δ*mamK* ($p < 0.0001$), and Δ*mad28* ($p = 0.0381$) compared to WT (Supplemental Table 9). (F) A one-way Kruskal-Wallis test comparing subchains per cell showed significant differences for Δ*mad10* ($p < 0.0001$), Δ*mad20* ($p < 0.0001$), Δ*mad25* ($p = 0.0073$), Δ*mad28* ($p = 0.0007$), and Δ*mamK* ($p < 0.0001$) relative to WT (Supplemental Table 9). (G-I) The percentage of cells exhibiting specific phenotypes, based on a count of 200 cells per strain. (G) The percentage of cells with crystals localized at the positive curvature of the cell. (H) The percentage of cells with magnetosomes displaying a chain phenotype. (I) The percentage of cells with all magnetosomes in the cell clustered together.

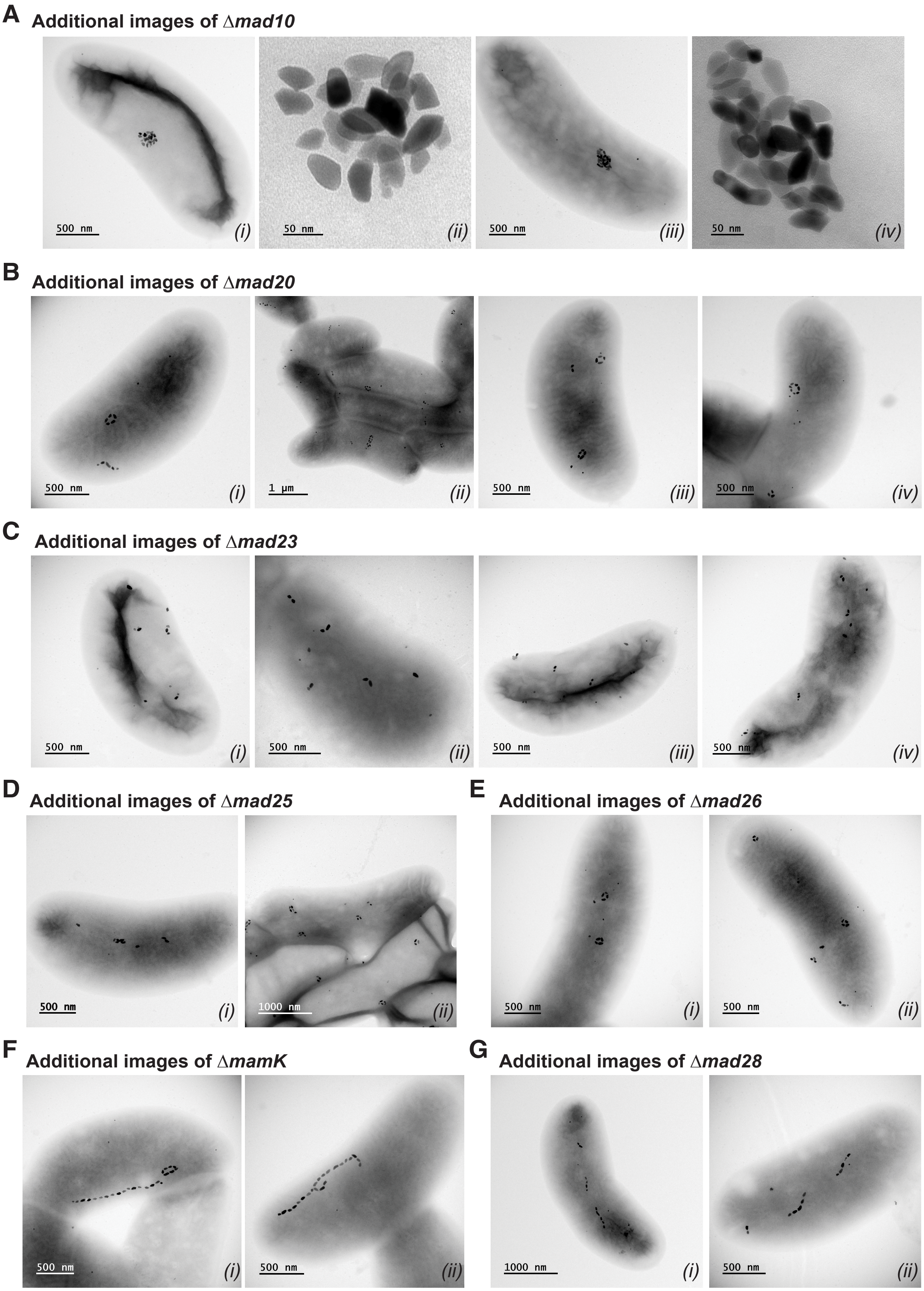
A
Additional images of Δmad10
500 nm
(i)
50 nm
(ii)
500 nm
(iii)
50 nm
(iv)
B
Additional images of Δmad20
500 nm
(i)
1 μm
(ii)
500 nm
(iii)
500 nm
(iv)
C
Additional images of Δmad23
500 nm
(i)
500 nm
(ii)
500 nm
(iii)
500 nm
(iv)
D
Additional images of Δmad25
500 nm
(i)
1000 nm
(ii)
E
Additional images of Δmad26
500 nm
(i)
500 nm
(ii)
F
Additional images of ΔmamK
500 nm
(i)
500 nm
(ii)
G
Additional images of Δmad28
1000 nm
(i)
500 nm
(ii)

Supplemental Figure 5. *Additional TEM images of gene deletions in RS-1 from this study.* (A) Additional TEM images of Δ*mad10* mutant. (A-*ii* and *iv*) are zoomed in images of the cluster of magnetosomes from A-*i* and *iii* respectively and illustrate the overlapping of magnetosomes. (B) Additional TEM images of Δ*mad20* mutant. These images illustrate fewer subchains in each cell and more crystals per subchain in each cell to complement the statistical data from Supplemental Figure 1. Perhaps rings form in this mutant because subchains are too long, similar to the Δ*mamK* phenotype. (C) Additional TEM images of Δ*mad23* in RS-1. These images illustrate the higher number of subchains in each cell and fewer crystals per subchain in each cell to complement the statistical data from Supplemental Figure 1. Subchains are more scattered in Δ*mad23*. (D) Additional TEM images of Δ*mad25* in RS-1. These images are to complement the statistical data from Supplemental Figure 1. (E) Additional TEM images of Δ*mad26* in RS-1. These images are to complement the statistical data from Supplemental Figure 1. Subchains are clustered in Δ*mad25* and Δ*mad26*. (F) Additional TEM images of Δ*mamK* in RS-1. These images illustrate the higher number of crystals in each cell and absence of subchains to complement the statistical data from Supplemental Figure 1. Additionally, the placement of the chain is not always in the center of the cell as seen in F-*ii*. (G) Additional TEM images of Δ*mad28* in RS-1. These images illustrate that chain placement is not at the positive curvature of the cell and to complement the statistical data from Supplemental Figure 1.

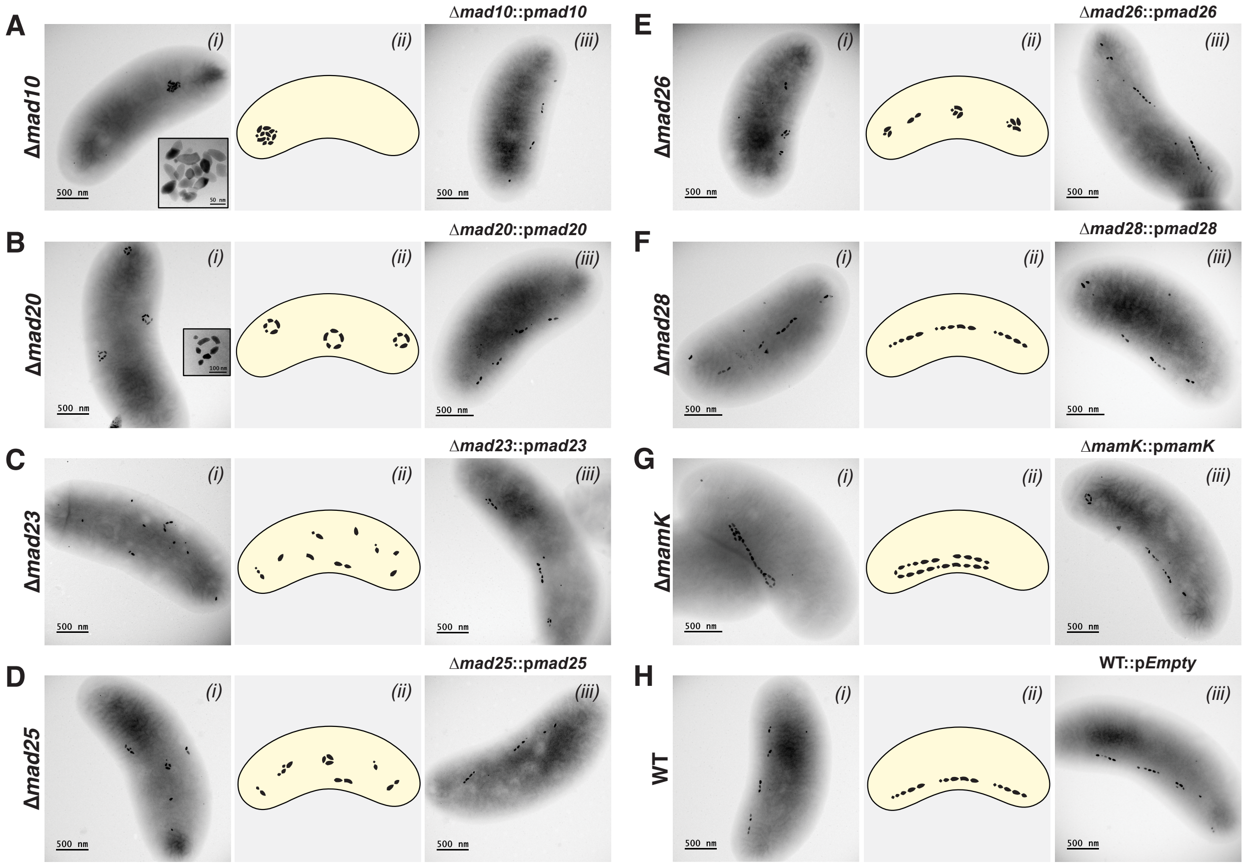

Supplemental Figure 6. *TEM images of complementations of deletion mutants*

(A-G) TEM images of deletion mutants and their respective complementation strains in RS-1. All complementations were done by expressing the gene on a plasmid. (H) TEM images of WT and a WT control with an empty plasmid.

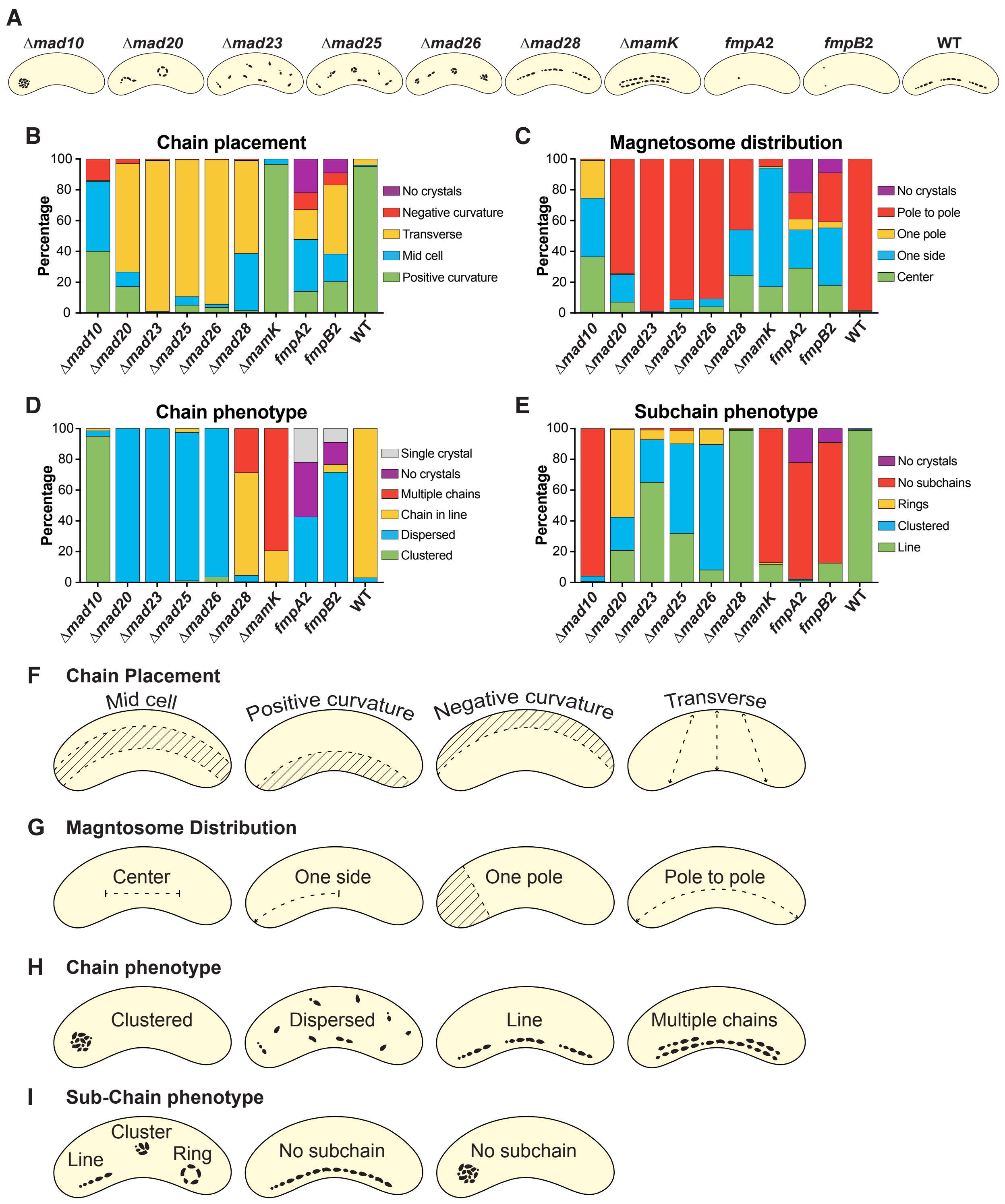

Supplemental Figure 7. *Additional mutant phenotypes and magnetosome chain and subchain categories*

(A) Illustrations of the phenotypes of the different mutants in this study. (B-E) Additional phenotype categories for all mutants. Over 200 cells were counted for each mutant. (F-I)

Categories used to calculate mutant phenotypes in Figure 4, Supplemental Figure 4, as well as (B-E) in this figure.

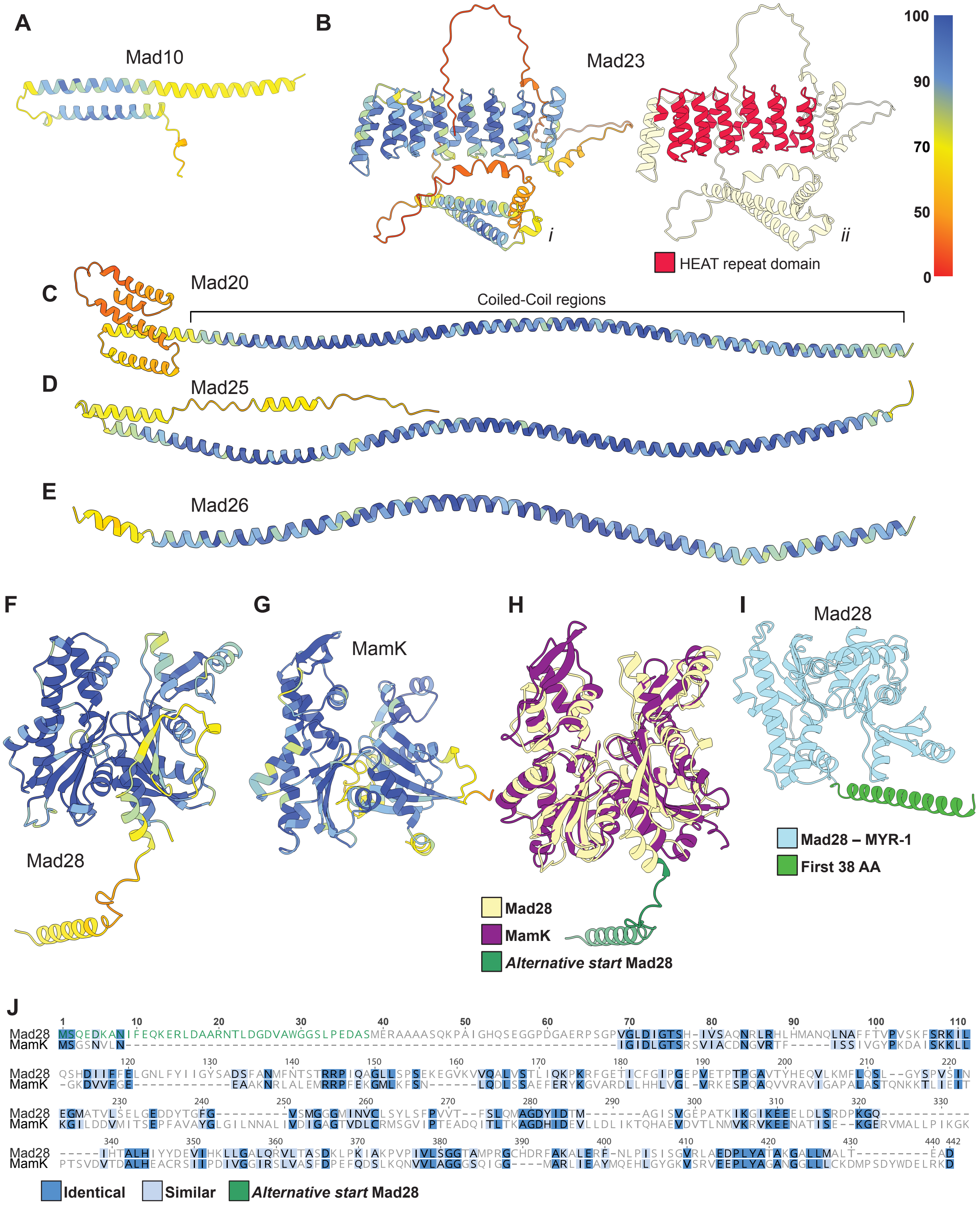

Supplemental Figure 8. *Predicted 3D Structures of magnetosome proteins deleted in RS-1 and alignments of MamK and Mad28*

(A-I) Predicted protein structures of Mad proteins and MamK in this study were made with Alphafold3[8] and visualized with ChimeraX[9]. 3D structures are colored according to Alphafold confidence values, with blue at 100% confident and red 0% confidence. (B-*ii*) Indicates the location of the HEAT domain (colored in red) in Mad23. (H) 3D alignment of protein structures of Mad28 and MamK from RS-1. Protein structures were made with alphafold3 and then a 3D alignment was preformed using ChimeraX[9]. Purple is the 3D predicted structure for MamK, yellow is the predicted structure for Mad28 and green is the part of the structure produced from the *mad28* alternative start codon. (I) Predicted 3D structure of Mad28 from MYR-1, a deep-branching MTB in the *Nitrospirota* phylum. The first 38 amino acids are highlighted in green to indicate structural similarity to the corresponding region in RS-1's Mad28. (J) 2D protein sequence alignment of MamK and Mad28 using MAFFT v7.490 plug-in on Geneious[4]. Blue highlighted residues show similarities between sequences and dashes represent gaps. Green letters show the sequence for the alternative start in Mad28.

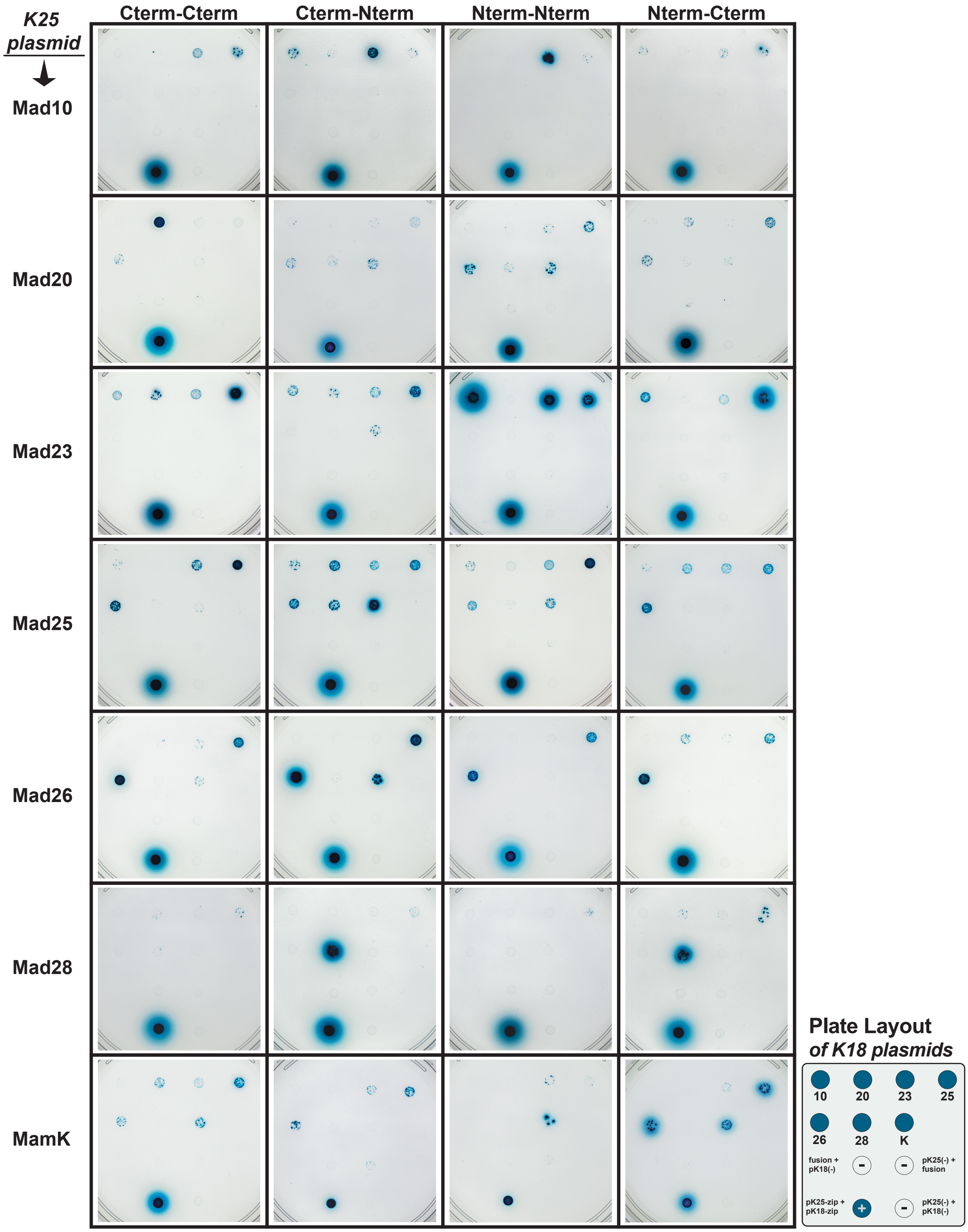

Supplemental Figure 9. *BACTH images of plates for all interactions.*

Image of all plates used to construct the table of the interactions in Figure 6. The illustration on the bottom right shows the layout for each plate and the controls used.

A

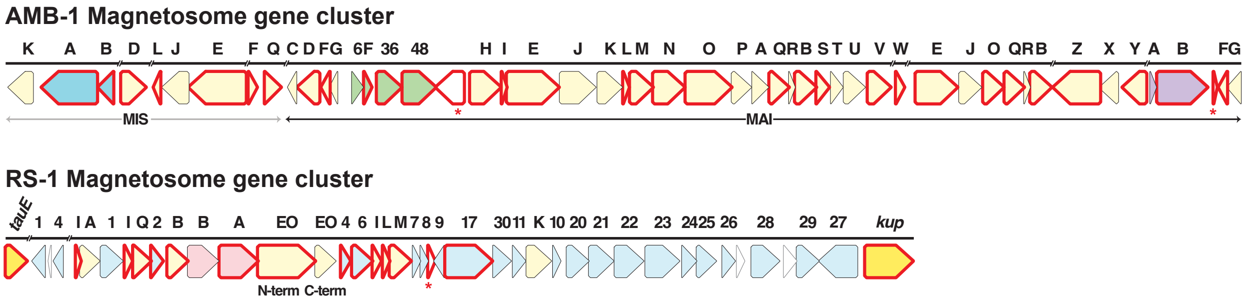

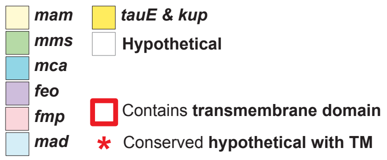

B Number of membrane genes in MGCs

	Total TM	Total genes	Proportion
RS-1 MGC	15	38	0.39
AMB-1 MGC	32	49	0.65

C

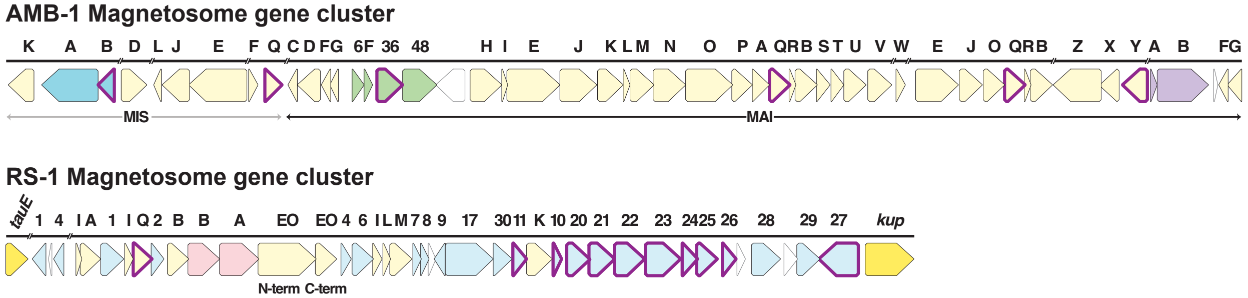

D Number of Coiled-coil genes in MGCs

	Total CC	Total genes	Proportion
RS-1 MGC	10	38	0.26
AMB-1 MGC	6	49	0.12

Supplemental Figure 10. *Magnetosome gene cluster comparison between AMB-1 and RS-1.* (A) Magnetosome gene clusters in AMB-1 and RS-1. Genes highlighted in red encode proteins with predicted transmembrane domains. (B) Table of the proportions of proteins with predicted transmembrane domains to all proteins encoded by the magnetosome gene clusters of AMB-1 and RS-1. DeepTMHMM[5] was used to gather transmembrane domain predictions. Hypothetical genes were not considered in the calculations for the proportions. (C) Magnetosome gene cluster of AMB-1 and RS-1 with genes encoding proteins with predicted coiled-coil domains highlighted in purple. (D) Table of the proportions of proteins with coiled-coil domains compared to all proteins in the MGC for AMB-1 and RS-1. DeepCoil[10] was used to obtain coiled-coil predictions. A threshold of 0.35 or greater significance with positive predictions for *a* and *d* core

positions was used to verify coiled-coil predictions. Hypothetical genes also were not considered in the calculations for the proportions of coiled-coil genes.

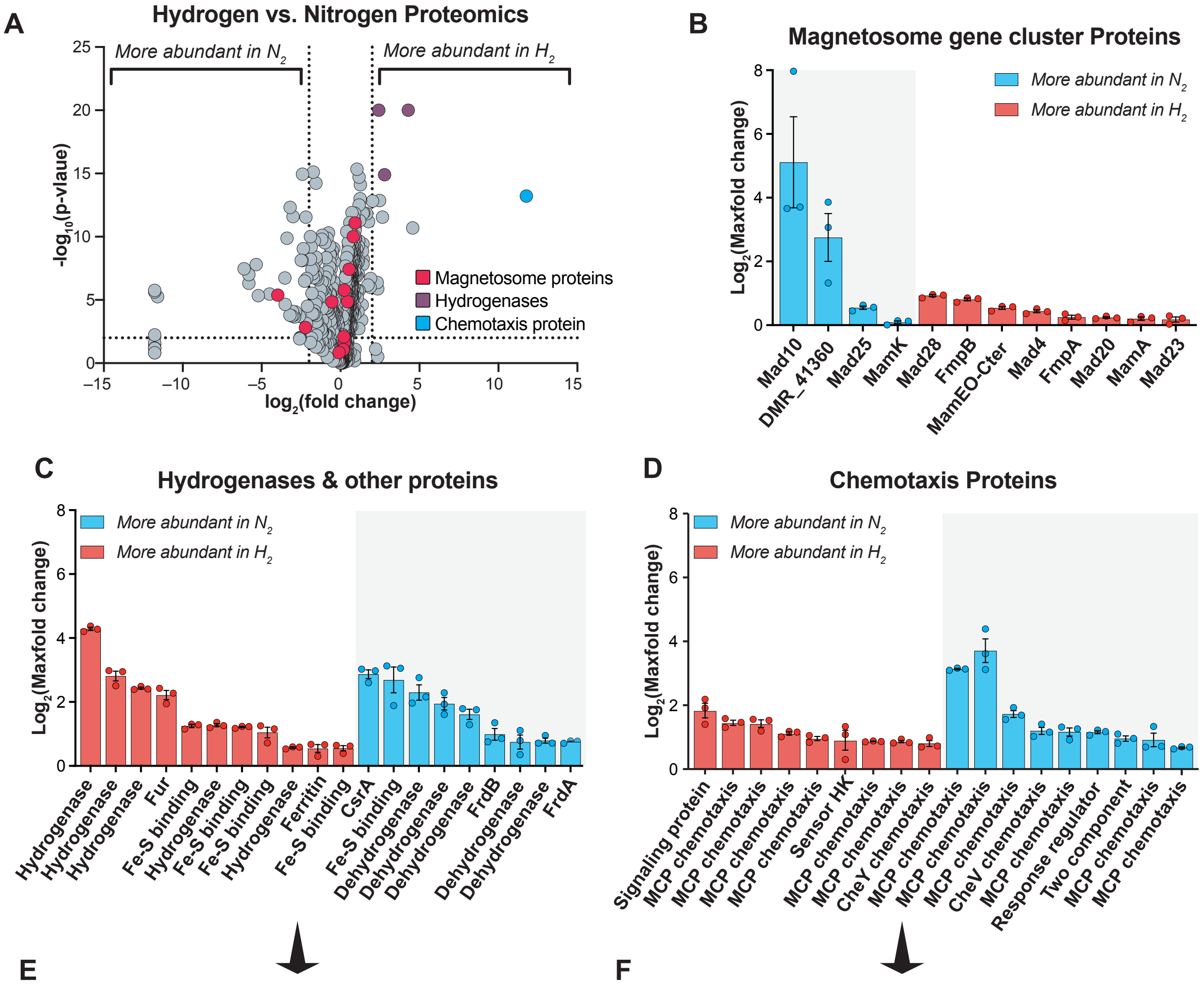

E

	Protein name	Highest
1	DMR_07840 Hydrogenase	Hydrogen
2	DMR_07830 Hydrogenase	Hydrogen
3	DMR_07850 Hydrogenase	Hydrogen
4	DMR_19790 Fur ferric uptake regulation	Hydrogen
5	DMR_01950 Fe-S binding	Hydrogen
6	DMR_01930 Hydrogenase	Hydrogen
7	DMR_01940 Fe-S binding	Hydrogen
8	DMR_20050 Fe-S binding	Hydrogen
9	DMR_12950 Hydrogenase	Hydrogen
10	DMR_17620 Ferritin	Hydrogen
11	DMR_42710 Fe-S binding	Hydrogen
12	DMR_31480 CsrA carbon storage regulator	Nitrogen
13	DMR_42500 Fe-S binding	Nitrogen
14	DMR_01890 Dehydrogenase	Nitrogen
15	DMR_16050 Dehydrogenase	Nitrogen
16	DMR_21490 Dehydrogenase	Nitrogen
17	DMR_05780 FrdB fumarate reductase	Nitrogen
18	DMR_19380 Dehydrogenase	Nitrogen
19	DMR_08200 Dehydrogenase	Nitrogen
20	DMR_34280 FrdA fumarate reductase	Nitrogen

F

	Protein name	Highest
1	DMR_09360 Signaling protein	Hydrogen
2	DMR_28950 Methyl-accepting chemotaxis	Hydrogen
3	DMR_00340 Methyl-accepting chemotaxis	Hydrogen
4	DMR_37860 Methyl-accepting chemotaxis	Hydrogen
5	DMR_27200 Methyl-accepting chemotaxis	Hydrogen
6	DMR_34160 Sensor histidine kinase	Hydrogen
7	DMR_38310 Methyl-accepting chemotaxis	Hydrogen
8	DMR_04320 Methyl-accepting chemotaxis	Hydrogen
9	DMR_23920 CheY chemotaxis	Hydrogen
10	DMR_26500 Methyl-accepting chemotaxis	Nitrogen
11	DMR_07950 Methyl-accepting chemotaxis	Nitrogen
12	DMR_29030 Methyl-accepting chemotaxis	Nitrogen
13	DMR_26640 CheV chemotaxis	Nitrogen
14	DMR_07730 Methyl-accepting chemotaxis	Nitrogen
15	DMR_41540 Response regulator	Nitrogen
16	DMR_45100 2-component sensor & regulator	Nitrogen
17	DMR_08750 Methyl-accepting chemotaxis	Nitrogen
18	DMR_27400 Methyl-accepting chemotaxis	Nitrogen

Supplemental Figure 11. *Additional proteomics data for hydrogen vs nitrogen.*

(A) A volcano plot showing the differential abundance analysis of the proteome between hydrogen and nitrogen conditions. Red dots indicate magnetosome genes, most of which fall between -2 and 2 $\log_2$ fold change, indicating no significant difference in abundance. Purple dots indicate hydrogenase proteins and blue dot indicates a chemotaxis protein, all of which are greater than 2 $\log_2$ fold more abundant in the hydrogen condition. Proteins with a peptide abundance of 0 in one condition may result in an infinite fold change. In such cases, the fold change was arbitrarily set to 3500. This is reflected in the volcano plots as a $\log_2$ value of -11.8. Additionally, p-values of 0 were arbitrarily assigned a $-\log_{10}$(p-value) of 20. (B) The maximum fold change of magnetosome proteins detected in cell lysates under both nitrogen and hydrogen conditions for three biological replicates. (C) and (D) Additional proteomic data comparing protein abundance in nitrogen versus hydrogen conditions, highlighting non-magnetosome proteins with maximum fold changes above 2. (B-D) The error bars represent +/- SEM of the biological replicates. Blue bars represent proteins more abundant in the nitrogen condition and orange bars represent proteins more abundant in hydrogen condition. (E) and (F) display the gene names and locus IDs corresponding to all bars in (C) and (D), respectively.

Supplemental Tables:

Phyla	Organiam	16S gene ID	Genome ID	Shape reference	MGC reference
Alpha	*Magnetospirillum magneticum* AMB-1	D17514.1	NCBI:txid342108	[11]	[12]
Alpha	*Magnetospirillum magnetotacticum* MS-1	IMG: 2647169949	NCBI:txid272627	[13]	[14]
Alpha	*Magnetospirillum caucaseum* SO-1	JX502622.2	NCBI:txid1244869	[15]	[15–17]
Alpha	*Magnetospirillum* ME-1	IMG: 2676550241	NCBI:txid1639348	[18]	[16,17,19]
Alpha	*Magnetospirillum* XM-1	KP966105.1	NCBI:txid1663591	[20]	[16,21]
Alpha	*Magnetospirillum marisnigri* SP-1	KC252630.3	NCBI:txid1285242	[15]	[17,22]
Alpha	*Magnetospirillum kuznetsovii* LBB-42	NR_180134.1	NCBI:txid2053833	[23]	[16,23]
Alpha	*Magnetospirillum gryphiswaldense* MSR-1	NR_121771.1	NCBI:txid431944	[24,25]	[26]
Alpha	*Magnetospirillum moscoviense* BB-1	KF712468.2	NCBI:txid1437059	[15]	[17,22]
Alpha	*Magnetospirillum* sp. LM-5	IMG: 2894467686	NCBI:txid2681466	[27]	[17]
Alpha	*Magnetospirillum* sp. SS-4	IMG: 2894461381	NCBI:txid2681465	[27]	[17]
Alpha	*Magnetospirillum* sp. UT-4	IMG: 2894472958	NCBI:txid2681467	[27]	[17]
Alpha	*Ca. Magneticavibrio boulderlitore* LM-1	JF490044.1	NCBI:txid1008128	[27]	[17]
Alpha	*Magnetospira* QH-2	EU675666.1	NCBI:txid1288970	[28]	[29]

Alpha	*Ca. Terasakiella magnetica* PR1	IMG: 2752957094	NCBI:txid1867952	30	30
Alpha	*Magnetovibrio blakemorei* MV-1	L06455.1	NCBI:txid28181	31	32
Gamma	Strain BW-2	HQ595728.1	NCBI:txid947515	33	34
Gamma	SHHR-1	KX344069.1	NCBI:txid1899433	35	16
Gamma	Strain SS-5	HQ595729.1	NCBI:txid947516	33	36
Eta	*Ca. Magnetococcus massalia* MO-1	EF643520.2	NCBI:txid451514	37	38
Eta	*Magnetococcus* MC-1	IMG: 640720050	NCBI:txid156889	39	40
Eta	*Magnetofaba australis* IT-1	JX534168.1	NCBI:txid1434232	41	41
Eta	*Ca. Magnetaquicoccus inordinatus* UR-1	IMG: 2881921666	NCBI:txid2496818	42	42
Desulfo-bacterota	*Ca. Magnetoglobus multicellularis*	IMG: 2559591426	NCBI:txid890399	43,44	45
Desulfo-bacterota	*Ca. Magnetananas rongchenensis* RPA	KF925363.1	NCBI:txid1463558	46	46
Desulfo-bacterota	*Ca. Magnetomorum* HK-1	IMG: 2648890082	NCBI:txid1509431	47	48
Desulfo-bacterota	*Desulfamplus magnetovallimortis* BW-1	JN252194.1	NCBI:txid1073250	49	50
Desulfo-bacterota	*Desulfovibrio magneticus* RS-1	NR_074958.1	NCBI:txid573370	51,52	53
Desulfo-bacterota	*Desulfovibrio* FSS-1	LC311577.1	NCBI:txid2730080	54	54
Desulfo-bacterota	*Delta Proteobacterium* FH-1	JF330268	NCBI:txid999117	50	50

Desulfo-bacterota	*Desulfonatronum* ML-1	HQ595725.1	NCBI:txid947513	55	50
Nitro-spirota	*Ca. Magnetoovum chiemensis* WYHC-5	OL423397.1	NCBI:txid1609970	56	56
Nitro-spirota	*Ca. Magnetobacterium casensis* MYR-1	MT703955.1	NCBI:txid1455061	57	58
Nitro-spirota	*Ca. Magnetobacterium bavaricum* TM-1	X71838.1	NCBI:txid29290	59	60
Nitro-spirota	*Ca. Nitrospirae* YQR-1	ON340538.1		16	16
Nitro-spirota	*Ca. Magnetomonas plexicatena* LBB-01	MK632185.1	CP049016.1	61	61
Nitro-spirota	*Ca. Magnetominusculus linsii* LBB-02	MK632186.1		61	61
Nitro-spirota	*Ca. Nitrospirae* MYC-10	ON342894.1		16	16
Nitro-spirota	*Ca. Magnetocorallium paracelense* XS-1	OQ281288.1	NCBI:txid3021403	62	62
Elusi-microbiota	*Ca. Liberimonas magnetica DUR002*	KY516811	GCA_020523885.1	6	6
Elusi-microbiota	*Ca. Obscuribacterium magneticum DUR003*	JQ369221	GCA_020523905.1	6	6
OP3	*Ca. Omnitrophus magneticus* SKK-01	JN412733.1	NCBI:txid174292	63	60

Supplemental Table 1. *Table of species and reference used for phylogenetic tree construction*

Strain	Description	Reference
AK80	WT	[64]
AK180	*fmpA2*^{Q418*}	[64]
AK182	*fmpB2*^{808delG}	[64]
AK268	WT, Δ*upp*	[65]
AK350	Δ*mad23*, Δ*upp*	This study
AK377	Δ*mad20*, Δ*upp*	This study
AK378	Δ*mad25*, Δ*upp*	This study
AK380	Δ*mad10*, Δ*upp*	This study
AK382	Δ*mad28*, Δ*upp*	This study
AK383	Δ*mad26*, Δ*upp*	This study
AK220	Δ*mamK*	This study

Supplemental Table 2. *Table of all strains used in this study*

Plasmid	Description	Backbone	Reference
PAK906	Complementation backbone with pNTP		[64]
PAK907	Complementation backbone with pmamA		[64]
pAK914	Deletion backbone with SacB only		[64]
pAK1127	Deletion backbone with Upp and SacB		[65]
pAK1452	mad10 deletion plasmid with Strep	pAK1127	This study
pAK1384	mad20 deletion plasmid with StrAB	pAK1127	This study
pAK1268	mad23 deletion with strAB for selection and upp counterselection (pCG116)	pAK1127	This study
pAK1386	mad25 deletion plasmid with StrAB	pAK1127	This study
pAK1387	mad26 deletion plasmid with StrAB	pAK1127	This study
pAK1388	mad28 deletion plasmid with StrAB	pAK1127	This study
pAK966	mamK deletion plasmid with strAB in pAK914	pAK914	This study
pAK1481	pAK906–mad10 Complementation	pAK906	This study
pAK1454	pAK906–mad20 complementation	pAK906	This study
pAK1507	pAK906–mad23 complementation	pAK906	This study
pAK1480	pAK907–mad25 Complementation	pAK907	This study
pAK1488	pAK907–mad26 Complementation	pAK907	This study

pAK1518	pAK907– mad28 Complementation (with alt Start)	pAK907	This study
pAK1489	pAK906–mamK Complementation	pAK906	This study
pAK318	pKT25		EUROMEDEX
pAK319	pKNT25		EUROMEDEX
pAK320	pUT18		EUROMEDEX
pAK321	pUT18C		EUROMEDEX
pAK322	pKT25-zip		EUROMEDEX
pAK323	pUT18C-zip		EUROMEDEX
pAK1525	KT25-mad10	pAK318	This study
pAK1526	mad10-KNT25	pAK319	This study
pAK1527	mad10-UT18	pAK320	This study
pAK1528	UT18C-mad10	pAK321	This study
pAK1529	KT25-mad20	pAK318	This study
pAK1530	mad20-KNT25	pAK319	This study
pAK1531	mad20-UT18	pAK320	This study
pAK1532	UT18C-mad20	pAK321	This study
pAK1541	KT25-mad23	pAK318	This study
pAK1542	mad23-KNT25	pAK319	This study
pAK1543	mad23-UT18	pAK320	This study
pAK1544	UT18C-mad23	pAK321	This study
pAK1549	KT25-mad25	pAK318	This study
pAK1550	mad25-KNT25	pAK319	This study
pAK1551	mad25-UT18	pAK320	This study
pAK1552	UT18C-mad25	pAK321	This study
pAK1553	KT25-mad26	pAK318	This study
pAK1554	mad26-KNT25	pAK319	This study
pAK1555	mad26-UT18	pAK320	This study
pAK1556	UT18C-mad26	pAK321	This study
pAK1557	KT25-mad28	pAK318	This study
pAK1558	mad28-KNT25	pAK319	This study
pAK1559	mad28-UT18	pAK320	This study
pAK1560	UT18C-mad28	pAK321	This study

pAK1561	KT25-mamK	pAK318	This study
pAK1562	mamK-KNT25	pAK319	This study
pAK1563	mamK-UT18	pAK320	This study
pAK1564	UT18C-mamK	pAK321	This study

Supplemental Table 3. *Table of all plasmids used in this study*

All of the plasmids used for this study and the previously published backbones used to generate the plasmids used for this research.

Primer	Sequence	Description
VR123-F	AAACGCAAAAGAAAATGCCGATATCCTATTG GCCTCTAGAGGAATTCCAGGATTCCCTCA	mad20 deletion upstream
VR124-R	GGATCCCCCATCCACTAAATTTAAATAGGATC CTCTGTCCTTAATCTTGGCC	mad20 deletion upstream
VR125-F	GGATCCTATTTAAATTTAGTGGATGGGGGAT CCAGGATCGCCAACAATGCCTG	mad20 deletion downstream
VR126-R	TGGCGGTCATGGTCTTTTCGGCGAGCTTCTC TAGAAGCTCCGCTTCAAGAACCAC	mad20 deletion downstream
VR127-F	AAACGCAAAAGAAAATGCCGATATCCTATTG GCCTCTAGACAAGTGGAGTTAGGGTGATT	mad25 deletion upstream
VR128-R	CATATGCCCATCCACTAAATTTAAATACATAT GGTCAGCTTTAAAAAGGGCCA	mad25 deletion upstream
VR129-F	CATATGTATTTAAATTTAGTGGATGGGCATAT GTGTTCGGCCATTGCCGTTAT	mad25 deletion downstream
VR130-R	TGGTGGCGGTCATGGTCTTTTCGGCGAGCTT CTCTAGACCTCGTCTCTCTAACACAAA	mad25 deletion downstream
VR131-F	AAACGCAAAAGAAAATGCCGATATCCTATTG GCCTCTAGACATCAAAAGCATGGAAGGGC	mad26 deletion upstream
VR132-R	GGATCCCCCATCCACTAAATTTAAATAGGATC CTTCGCGCTGCATTCCGTCCA	mad26 deletion upstream
VR133-F	GGATCCTATTTAAATTTAGTGGATGGGGGAT CCACTATTGCCTTGAATAAATG	mad26 deletion downstream
VR134-R	TGGTGGCGGTCATGGTCTTTTCGGCGAGCTT CTCTAGAGCATCCAAGCGTTCTTTTTG	mad26 deletion downstream

VR135-F	AAACGCAAAAGAAAATGCCGATATCCTATTG GCCTCTAGAAACGGTTTACAGCGGATACA	mad28 deletion upstream
VR136-R	GGATCCCCCATCCACTAAATTTAAATAGGATC CGCATCCAAGCGTTCTTTTTG	mad28 deletion upstream
VR137-F	GGATCCTATTTAAATTTAGTGGATGGGGGAT CCGGCCCTGACCGAGGCGGATT	mad28 deletion downstream
VR138-R	TGGCGGTCATGGTCTTTTCGGCGAGCTTCTC TAGACAAAATGGAGATTGGAGGAA	mad28 deletion downstream
VR147-F	AGGCGGGCATGGCCAAGATTAAGGACAGAG GATCCCTGCTAAAGGAAGCGGAACACG	Add StrAB-mad20-F
VR148-R	ATTCGCTATCAGGCATTGTTGGCGATCCTGG ATCCCTAGTATGACGTCTGTCGCACCT	Add StrAB-mad20-R
VR149-F	AGGGGCCATGGCCCTTTTTAAAGCTGACCAT ATGCTGCTAAAGGAAGCGGAACACG	Add StrAB-mad25-F
VR150-R	GAAAAAGCATAACGGCAATGGCCGAACACAT ATGCTAGTATGACGTCTGTCGCACCT	Add StrAB-mad25-R
VR151-F	GGGCAAACACTCCCGGTCGATTCCGGACAG GATCCCTGCTAAAGGAAGCGGAACACG	Add StrAB-mad26-F
VR152-R	ATCACAGTTCATTTATTCAAGGCAATAGTGGA TCCCTAGTATGACGTCTGTCGCACCT	Add StrAB-mad26-R
VR153-F	ATATTCGAGCAAAAAGAACGCTTGGATGCGG ATCCCTGCTAAAGGAAGCGGAACACG	Add StrAB-mad28-F
VR154-R	GGTATGGCTAATCCGCCTCGGTCAGGGCCG GATCCCTAGTATGACGTCTGTCGCACCT	Add StrAB-mad28-R
VR319-F	GTCATGGTCTTTTCGGCGAGCTTCTCTAGAA TCTCCAAAAAGTTGCTGGGC	mad10 deletion upstream
VR320-R	CCCATCCACTAAATTTAAATAGGATCCTCTTC CATGGCGTCCTCCG	mad10 deletion upstream
VR321-F	TATTTAAATTTAGTGGATGGGGGATCCCCAG TGCGCAGCCGGCCTGA	mad10 deletion downstream
VR322-R	GAAAATGCCGATATCCTATTGGCCTCTAGAT CGCGGCCTGGGCC	mad10 deletion downstream

VR323-F	CGACAATGCGCGGAGGACGCCATGGAAGAG CTGCTAAAGGAAGCGGAACAC	Add StrAB-mad10-F
VR324-R	GAGATGATCTCAGGCCGGCTGCGCACTGGG CTAGTATGACGTCTGTCGCACC	Add StrAB-mad10-R
VR277-F	AGACAGGATGAGGATCGTTTCGCGTCGACTG ATCATCTCCCGGCGGG	mad20 complementation in pAK906
VR278-R	GGGAATTCGAGCTCGGTACCCGGGGATCCT GCGCCGCTCCTGGATTC	mad20 complementation in pAK906
CG220-R	GAGCTCGGTACCCGGGGATCCTCTAGAACTT GCTTTTCCGCCGTCATAC	mad23 complementation in pAK906
CG221-F	CAAGAGACAGGATGAGGATCGTTTCGCGATG GAAGACGCCATGAGCTC	mad23 complementation in pAK906
VR335-F	AAGCCAAGAAAAACGTCGCCAACGTCGACTA TGGCCCTTTTTAAAGCTGACG	mad25 complementation in pAK907
VR336-R	GGGAATTCGAGCTCGGTACCCGGGGATCCT CTACCCGTCTGCGACGTC	mad25 complementation in pAK907
VR337-F	AGACAGGATGAGGATCGTTTCGCGTCGACTA TGGAAGAAAATACCCGCTACA	mad10 complementation in pAK906
VR338R	GGGAATTCGAGCTCGGTACCCGGGGATCCT TCAGGCCGGCTGCG	mad10 complementation in pAK906
VR355-F	AAGCCAAGAAAAACGTCGCCAACGTCGACTA TGGACGGAATGCAGCGC	mad26 complementation in pAK907

VR356-R	GGGAATTCGAGCTCGGTACCCGGGGATCCT TCATTTATTCAAGGCAATAGTCAGT	mad26 complementation in pAK907
VR357-F	AGACAGGATGAGGATCGTTTCGCGTCGACTA TGTCCGGAAGCAACGTGC	mamK complementation in pAK906
VR358-R	GGGAATTCGAGCTCGGTACCCGGGGATCCT TTAATCCTTTCGCAGCTCGTC	mamK complementation in pAK906
VR375-F	AAGCCAAGAAAAACGTCGCCAACGTCGACTA CAGCTCTGGCGCTCTTG	mad28 complementation in pAK907 (with pmad28 & alt-start)
VR372-R	GGGAATTCGAGCTCGGTACCCGGGGATCCT CTAATCCGCCTCGGTCAGG	mad28 complementation in pAK907 (with pmad28 & alt-start)
VR390-F	CGGGCTGCAGGGTCGACTCTAGAGGATCCC ATGGAAGAAAATACCCGCTACA	KT25_mad10
VR391-R	TCACGACGTTGTAAAACGACGGCCGAATTCT CAGGCCGGCTGCG	KT25_mad10
VR392-F	ACAGCTATGACCATGATTACGCCAAGCTTGA TGGAAGAAAATACCCGCTACA	KNT25_mad10-noStop
VR393-R	CCGGGGATCCTCTAGAGTCGACCTGCAGGC GGCCGGCTGCGCA	KNT25_mad10-noStop
VR394-F	ACAGCTATGACCATGATTACGCCAAGCTTGA TGGAAGAAAATACCCGCTACA	UT18_mad10-NoStop
VR395-R	CCGGGGATCCTCTAGAGTCGACCTGCAGGC GGCCGGCTGCGCA	UT18_mad10-NoStop
VR396-F	CGCCACTGCAGGTCGACTCTAGAGGATCCCA TGGAAGAAAATACCCGCTACA	UT18C_mad10
VR397-R	TAGTTATATCGATGAATTCGAGCTCGGTACTC AGGCCGGCTGCG	UT18C_mad10
VR398-F	CGGGCTGCAGGGTCGACTCTAGAGGATCCC ATGGCCAAGATTAAGGACAGACT	KT25_mad20

VR399-R	TCACGACGTTGTAAAACGACGGCCGAATTCT CAGGCATTGTTGGCGATCC	KT25_mad20
VR400-F	ACAGCTATGACCATGATTACGCCAAGCTTGA TGGCCAAGATTAAGGACAGACT	KNT25_mad20-noStop
VR401-R	CCGGGGATCCTCTAGAGTCGACCTGCAGGC GGCATTGTTGGCGATCCTCT	KNT25_mad20-noStop
VR402-F	ACAGCTATGACCATGATTACGCCAAGCTTGA TGGCCAAGATTAAGGACAGACT	UT18_mad20-NoStop
VR403-R	CCGGGGATCCTCTAGAGTCGACCTGCAGGC GGCATTGTTGGCGATCCTCT	UT18_mad20-NoStop
VR404-F	CGCCACTGCAGGTCGACTCTAGAGGATCCCA TGGCCAAGATTAAGGACAGACT	UT18C_mad20
VR405-R	TAGTTATATCGATGAATTCGAGCTCGGTACTC AGGCATTGTTGGCGATCC	UT18C_mad20
VR422-F	CGGGCTGCAGGGTCGACTCTAGAGGATCCC ATGGAAGACGCCATGAGCTC	KT25_mad23
VR423-R	TCACGACGTTGTAAAACGACGGCCGAATTCC TAACTCCACTTGTAGCGCA	KT25_mad23
VR424-F	ACAGCTATGACCATGATTACGCCAAGCTTGA TGGAAGACGCCATGAGCTC	KNT25_mad23-noStop
VR425-R	CCGGGGATCCTCTAGAGTCGACCTGCAGGC ACTCCACTTGTAGCGCATGC	KNT25_mad23-noStop
VR426-F	ACAGCTATGACCATGATTACGCCAAGCTTGA TGGAAGACGCCATGAGCTC	UT18_mad23-NoStop
VR427-R	CCGGGGATCCTCTAGAGTCGACCTGCAGGC ACTCCACTTGTAGCGCATGC	UT18_mad23-NoStop
VR428-F	CGCCACTGCAGGTCGACTCTAGAGGATCCCA TGGAAGACGCCATGAGCTC	UT18C_mad23
VR429-R	TAGTTATATCGATGAATTCGAGCTCGGTACCT AACTCCACTTGTAGCGCA	UT18C_mad23
VR438-F	CGGGCTGCAGGGTCGACTCTAGAGGATCCC ATGGCCCTTTTTAAAGCTGACG	KT25_mad25

VR439-R	TCACGACGTTGTAAAACGACGGCCGAATTCCTACCCGTCTGCGACGTC	KT25_mad25
VR440-F	ACAGCTATGACCATGATTACGCCAAGCTTGATGGCCCTTTTTAAAGCTGACG	KNT25_mad25-noStop
VR441-R	CCGGGGATCCTCTAGAGTCGACCTGCAGGCCCCGTCTGCGACGTCG	KNT25_mad25-noStop
VR442-F	ACAGCTATGACCATGATTACGCCAAGCTTGATGGCCCTTTTTAAAGCTGACG	UT18_mad25-NoStop
VR443-R	CCGGGGATCCTCTAGAGTCGACCTGCAGGCCCCGTCTGCGACGTCG	UT18_mad25-NoStop
VR444-F	CGCCACTGCAGGTCGACTCTAGAGGATCCCATGGCCCTTTTTAAAGCTGACG	UT18C_mad25
VR445-R	TAGTTATATCGATGAATTCGAGCTCGGTACCTACCCGTCTGCGACGTC	UT18C_mad25
VR446-F	CGGGCTGCAGGGTCGACTCTAGAGGATCCCATGGACGGAATGCAGCGC	KT25_mad26
VR447-R	TCACGACGTTGTAAAACGACGGCCGAATTCTCATTTATTCAAGGCAATAGTCAGT	KT25_mad26
VR448-F	ACAGCTATGACCATGATTACGCCAAGCTTGATGGACGGAATGCAGCGC	KNT25_mad26-noStop
VR449-R	CCGGGGATCCTCTAGAGTCGACCTGCAGGCTTTATTCAAGGCAATAGTCAGTTCA	KNT25_mad26-noStop
VR450-F	ACAGCTATGACCATGATTACGCCAAGCTTGATGGACGGAATGCAGCGC	UT18_mad26-NoStop
VR451-R	CCGGGGATCCTCTAGAGTCGACCTGCAGGCTTTATTCAAGGCAATAGTCAGTTCA	UT18_mad26-NoStop
VR452-F	CGCCACTGCAGGTCGACTCTAGAGGATCCCATGGACGGAATGCAGCGC	UT18C_mad26
VR453-R	TAGTTATATCGATGAATTCGAGCTCGGTACTCATTTATTCAAGGCAATAGTCAGT	UT18C_mad26
VR454-F	CGGGCTGCAGGGTCGACTCTAGAGGATCCCATGTCCCAAGAGGATAAGGCG	KT25_mad28

VR455-R	TCACGACGTTGTAAAACGACGGCCGAATTCC TAATCCGCCTCGGTCAGG	KT25_mad28
VR456-F	ACAGCTATGACCATGATTACGCCAAGCTTGA TGTCCCAAGAGGATAAGGCG	KNT25_mad28-noStop
VR457-R	CCGGGGATCCTCTAGAGTCGACCTGCAGGC ATCCGCCTCGGTCAGGG	KNT25_mad28-noStop
VR458-F	ACAGCTATGACCATGATTACGCCAAGCTTGA TGTCCCAAGAGGATAAGGCG	UT18_mad28-NoStop
VR459-R	CCGGGGATCCTCTAGAGTCGACCTGCAGGC ATCCGCCTCGGTCAGGG	UT18_mad28-NoStop
VR460-F	CGCCACTGCAGGTCGACTCTAGAGGATCCCA TGTCCCAAGAGGATAAGGCG	UT18C_mad28
VR461-R	TAGTTATATCGATGAATTCGAGCTCGGTACCT AATCCGCCTCGGTCAGG	UT18C_mad28
VR462-F	CGGGCTGCAGGGTCGACTCTAGAGGATCCC ATGTCCGGAAGCAACGTGC	KT25_mamK
VR463-R	TCACGACGTTGTAAAACGACGGCCGAATTCT TAATCCTTTCGCAGCTCGTC	KT25_mamK
VR464-F	ACAGCTATGACCATGATTACGCCAAGCTTGA TGTCCGGAAGCAACGTGC	KNT25_mamK-noStop
VR465-R	CCGGGGATCCTCTAGAGTCGACCTGCAGGC ATCCTTTCGCAGCTCGTCC	KNT25_mamK-noStop
VR466-F	ACAGCTATGACCATGATTACGCCAAGCTTGA TGTCCGGAAGCAACGTGC	UT18_mamK-NoStop
VR467-R	CCGGGGATCCTCTAGAGTCGACCTGCAGGC ATCCTTTCGCAGCTCGTCC	UT18_mamK-NoStop
VR468-F	CGCCACTGCAGGTCGACTCTAGAGGATCCCA TGTCCGGAAGCAACGTGC	UT18C_mamK
VR469-R	TAGTTATATCGATGAATTCGAGCTCGGTACTT AATCCTTTCGCAGCTCGTC	UT18C_mamK

Supplemental Table 4. *Table of all primers used in this study*

		**Compared with:**	**Welch's T-Test** (two-tailed)	
**Figure**	**Strain/ Condition**	**Strain/ Condition**	**pValue**	**Significance**
Figure 2B	Hydrogen C_{mag}	Nitrogen C_{mag}	0.0009	***
Figure 2D *inlet*	Hydrogen (doubling time)	Nitrogen (doubling time)	0.0093	**

Supplemental Table 5. *Table of all statistical tests used for Figure 2 in this study*

		**Compared with:**	**Mann-Whitney U test** (two-tailed)		**Welch's T-Test** (two-tailed)	
**Figure**	**Strain/ Condition**	**Strain/ Condition**	**pValue**	**Significance**	**pValue**	**Significance**
Figure 3C	Early	Late	<0.0001	****	<0.0001	****
Figure 3D	Early	Late	<0.0001	****	<0.0001	****
Figure 3E	Early	Late	<0.0001	****	<0.0001	****
Figure 3I	Immature crystals	Mature crystals	---	---	0.0006	***

Supplemental Table 6. *Table of all statistical tests used for Figure 3 in this study*

		**Compared with:**	**One way ANOVA test**		**Mann-Whitney U test** (two-tailed)	
**Figure**	**Strain/ Condition**	**Strain/ Condition**	**pValue**	**Significance**	**pValue**	**Significance**
Figure 4D	*fmpA*	WT	<0.0001	****	<0.0001	****
Figure 4D	*fmpB*	WT	<0.0001	****	<0.0001	****
Figure 4E	*fmpA*	WT	<0.0001	****	<0.0001	****
Figure 4E	*fmpB*	WT	<0.0001	****	<0.0001	****
Figure 4F	*fmpA*	WT	<0.0001	****	<0.0001	****

Figure 4F	*fmpB*	WT	<0.0001	****	<0.0001	****

Supplemental Table 7. *Table of all statistical tests used for Figure 4 in this study*

		Compared with:	One way ANOVA test		Mann-Whitney U test (two-tailed)	
**Figure**	**Strain/ Condition**	**Strain/ Condition**	**pValue**	**Significance**	**pValue**	**Significance**
Figure 5N*i*	Δ*mamK*	WT	<0.0001	****	<0.0001	****
Figure 5N*i*	Δ*mad28*	WT	0.9378	ns	0.4697	ns
Figure 5N*ii*	Δ*mamK*	WT	<0.0001	****	<0.0001	****
Figure 5N*ii*	Δ*mad28*	WT	0.0703	ns	0.0053	**

Supplemental Table 8. *Table of all statistical tests used for Figure 5 in this study*

		Compared with:	One way ANOVA test	
**Supplemental Figure**	**Strain/ Condition**	**Strain/ Condition**	**pValue**	**Significance**
Supplemental Figure 4B	Δ*mad10*	WT	<0.0001	****
Supplemental Figure 4B	Δ*mad20*	WT	0.0002	***
Supplemental Figure 4B	Δ*mad23*	WT	<0.0001	****
Supplemental Figure 4B	Δ*mad25*	WT	0.0030	**
Supplemental Figure 4B	Δ*mad26*	WT	0.0005	***
Supplemental Figure 4B	Δ*mad28*	WT	<0.0001	****
Supplemental Figure 4B	Δ*mamK*	WT	0.0030	**

		Compared with:	One way ANOVA test	
**Supplemental Figure**	**Strain/ Condition**	**Strain/ Condition**	**pValue**	**Significance**
Supplemental Figure 4C	Δ*mad10::pMad10*	Δ*mad10::pEmpty*	<0.0001	****
Supplemental Figure 4C	Δ*mad20::pMad20*	Δ*mad20:: pEmpty*	<0.0001	****

Supplemental Figure 4C	Δ*mad23::pMad23*	Δ*mad23:: pEmpty*	<0.0001	****
Supplemental Figure 4C	Δ*mad25::pMad25*	Δ*mad25:: pEmpty*	0.0002	***
Supplemental Figure 4C	Δ*mad26::pMad26*	Δ*mad26:: pEmpty*	<0.0001	****
Supplemental Figure 4C	Δ*mad28::pMad28*	Δ*mad28:: pEmpty*	<0.0001	****
Supplemental Figure 4C	Δ*mamK::pMamK*	Δ*mamK:: pEmpty*	<0.0001	****

		**Compared with:**	**One way ANOVA test**		**Mann-Whitney U test** (two-tailed)	
**Supplemental Figure**	**Strain/ Condition**	**Strain/ Condition**	**pValue**	**Significance**	**pValue**	**Significance**
Supplemental Figure 4D	Δ*mad10*	WT	0.1364	ns	0.0005	***
Supplemental Figure 4D	Δ*mad20*	WT	0.7606	ns	0.3754	ns
Supplemental Figure 4D	Δ*mad23*	WT	0.7606	ns	0.5328	ns
Supplemental Figure 4D	Δ*mad25*	WT	0.3521	ns	0.0147	*
Supplemental Figure 4D	Δ*mad26*	WT	0.4881	ns	0.111	ns
Supplemental Figure 4D	Δ*mad28*	WT	0.7606	ns	0.3365	ns
Supplemental Figure 4D	Δ*mamK*	WT	<0.0001	****	<0.0001	****

		**Compared with:**	**One way ANOVA test**	
**Supplemental Figure**	**Strain/Condition**	**Strain/Condition**	**pValue**	**Significance**

Supplemental Figure 4E	Δ*mad10*	WT	<0.0001	****
Supplemental Figure 4E	Δ*mad20*	WT	0.0001	***
Supplemental Figure 4E	Δ*mad23*	WT	0.5127	ns
Supplemental Figure 4E	Δ*mad25*	WT	0.9895	ns
Supplemental Figure 4E	Δ*mad26*	WT	0.0953	ns
Supplemental Figure 4E	Δ*mad28*	WT	0.0381	*
Supplemental Figure 4E	Δ*mamK*	WT	<0.0001	****

		**Compared with:**	**Kruskal-Wallis test** (one way test)	
**Supplemental Figure**	**Strain/Condition**	**Strain/Condition**	**pValue**	**Significance**
Supplemental Figure 4F	Δ*mad10*	WT	<0.0001	****
Supplemental Figure 4F	Δ*mad20*	WT	<0.0001	****
Supplemental Figure 4F	Δ*mad23*	WT	0.2744	ns
Supplemental Figure 4F	Δ*mad25*	WT	0.0073	**
Supplemental Figure 4F	Δ*mad26*	WT	0.0805	ns
Supplemental Figure 4F	Δ*mad28*	WT	0.0007	***
Supplemental Figure 4F	Δ*mamK*	WT	<0.0001	****

Supplemental Table 9. *Tables of all statistical tests used for Supplemental Figure 4 in this study*

Supplementary References:

1. Liu, Y., Van Den Ent, F. & Löwe, J. Filament structure and subcellular organization of the bacterial intermediate filament–like protein crescentin. *Proc. Natl. Acad. Sci.* **121**, e2309984121 (2024).
2. Pohl, A. *et al.* Decoding Biomineralization: Interaction of a Mad10-Derived Peptide with Magnetite Thin Films. *Nano Lett.* **19**, 8207–8215 (2019).
3. Paysan-Lafosse, T. *et al.* InterPro in 2022. *Nucleic Acids Res.* **51**, D418–D427 (2023).
4. Kearse, M. *et al.* Geneious Basic: An integrated and extendable desktop software platform for the organization and analysis of sequence data. *Bioinformatics* **28**, 1647–1649 (2012).
5. Hallgren, J. *et al.* DeepTMHMM predicts alpha and beta transmembrane proteins using deep neural networks. Preprint at https://doi.org/10.1101/2022.04.08.487609 (2022).
6. Uzun, M. *et al.* Recovery and genome reconstruction of novel magnetotactic Elusimicrobiota from bog soil. *ISME J.* **17**, 204–214 (2023).
7. Gilchrist, C. L. M. & Chooi, Y.-H. clinker & clustermap.js: automatic generation of gene cluster comparison figures. *Bioinformatics* **37**, 2473–2475 (2021).
8. Mirdita, M. *et al.* ColabFold: making protein folding accessible to all. *Nat. Methods* **19**, 679–682 (2022).
9. Meng, E. C. *et al.* UCSF ChimeraX: Tools for structure building and analysis. *Protein Sci. Publ. Protein Soc.* **32**, e4792 (2023).
10. Ludwiczak, J., Winski, A., Szczepaniak, K., Alva, V. & Dunin-Horkawicz, S. DeepCoil—a fast and accurate prediction of coiled-coil domains in protein sequences. *Bioinformatics* **35**, 2790–2795 (2019).

11. Li, J. & Pan, Y. Environmental Factors Affect Magnetite Magnetosome Synthesis in Magnetospirillum magneticum AMB-1: Implications for Biologically Controlled Mineralization. *Geomicrobiol. J.* **29**, 362–373 (2012).
12. Matsunaga, T. *et al.* Complete genome sequence of the facultative anaerobic magnetotactic bacterium Magnetospirillum sp. strain AMB-1. *DNA Res. Int. J. Rapid Publ. Rep. Genes Genomes* **12**, 157–166 (2005).
13. Maratea, D. & Blakemore, R. P. Aquaspirillum magnetotacticum sp. nov., a Magnetic Spirillum. *Int. J. Syst. Evol. Microbiol.* **31**, 452–455 (1981).
14. Smalley, M. D., Marinov, G. K., Bertani, L. E. & DeSalvo, G. Genome Sequence of Magnetospirillum magnetotacticum Strain MS-1. *Genome Announc.* **3**, e00233-15 (2015).
15. Dziuba, M. *et al.* Magnetospirillum caucaseum sp. nov., Magnetospirillum marisnigri sp. nov. and Magnetospirillum moscoviense sp. nov., freshwater magnetotactic bacteria isolated from three distinct geographical locations in European Russia. *Int. J. Syst. Evol. Microbiol.* **66**, 2069–2077 (2016).
16. Liu, P. *et al.* Key gene networks that control magnetosome biomineralization in magnetotactic bacteria. *Natl. Sci. Rev.* **10**, nwac238 (2023).
17. Monteil, C. L. *et al.* Repeated horizontal gene transfers triggered parallel evolution of magnetotaxis in two evolutionary divergent lineages of magnetotactic bacteria. *ISME J.* **14**, 1783–1794 (2020).
18. Ke, L. *et al.* Characteristics and optimised fermentation of a novel magnetotactic bacterium, Magnetospirillum sp. ME-1. *FEMS Microbiol. Lett.* **365**, fny052 (2018).

19. Ke, L., Liu, P., Liu, S. & Gao, M. Complete Genome Sequence of Magnetospirillum sp. ME-1, a Novel Magnetotactic Bacterium Isolated from East Lake, Wuhan, China. *Genome Announc.* **5**, e00485-17 (2017).

20. Wang, Y. *et al.* Characterizing and optimizing magnetosome production of Magnetospirillum sp. XM-1 isolated from Xi'an City Moat, China. *FEMS Microbiol. Lett.* **362**, fnv167 (2015).

21. Wang, Y. *et al.* Complete Genome Sequence of Magnetospirillum sp. Strain XM-1, Isolated from the Xi'an City Moat, China. *Genome Announc.* **4**, e01171-16 (2016).

22. Koziaeva, V. V. *et al.* Draft Genome Sequences of Two Magnetotactic Bacteria, Magnetospirillum moscoviense BB-1 and Magnetospirillum marisnigri SP-1. *Genome Announc.* **4**, e00814-16 (2016).

23. Koziaeva, V. V. *et al.* Magnetospirillum kuznetsovii sp. nov., a novel magnetotactic bacterium isolated from a lake in the Moscow region. *Int. J. Syst. Evol. Microbiol.* **69**, 1953–1959 (2019).

24. Fdez-Gubieda, M. L. *et al.* Magnetite Biomineralization in Magnetospirillum gryphiswaldense: Time-Resolved Magnetic and Structural Studies. *ACS Nano* **7**, 3297–3305 (2013).

25. Schleifer, K. H. *et al.* The Genus *Magnetospirillum* gen. nov. Description of *Magnetospirillum gryphiswaldense* sp. nov. and Transfer of *Aquaspirillum magnetotacticum* to *Magnetospirillum magnetotacticum* comb. nov. *Syst. Appl. Microbiol.* **14**, 379–385 (1991).

26. Wang, X. *et al.* Complete Genome Sequence of Magnetospirillum gryphiswaldense MSR-1. *Genome Announc.* **2**, e00171-14 (2014).

27. Lefèvre, C. T. *et al.* Insight into the Evolution of Magnetotaxis in Magnetospirillum spp., Based on *mam* Gene Phylogeny. *Appl. Environ. Microbiol.* **78**, 7238–7248 (2012).
28. Zhu, K. *et al.* Isolation and characterization of a marine magnetotactic spirillum axenic culture QH-2 from an intertidal zone of the China Sea. *Res. Microbiol.* **161**, 276–283 (2010).
29. Ji, B. *et al.* Comparative genomic analysis provides insights into the evolution and niche adaptation of marine Magnetospira sp. QH-2 strain. *Environ. Microbiol.* **16**, 525–544 (2014).
30. Monteil, C. L. *et al.* Genomic study of a novel magnetotactic Alphaproteobacteria uncovers the multiple ancestry of magnetotaxis. *Environ. Microbiol.* **20**, 4415–4430 (2018).
31. Bazylinski, D. A. *et al.* Magnetovibrio blakemorei gen. nov., sp. nov., a magnetotactic bacterium (Alphaproteobacteria: Rhodospirillaceae) isolated from a salt marsh. *Int. J. Syst. Evol. Microbiol.* **63**, 1824–1833 (2013).
32. Trubitsyn, D. *et al.* Draft Genome Sequence of Magnetovibrio blakemorei Strain MV-1, a Marine Vibrioid Magnetotactic Bacterium. *Genome Announc.* **4**, e01330-16 (2016).
33. Lefèvre, C. T. *et al.* Novel magnetite-producing magnetotactic bacteria belonging to the Gammaproteobacteria. *ISME J.* **6**, 440–450 (2012).
34. Geurink, C. *et al.* Complete Genome Sequence of Strain BW-2, a Magnetotactic Gammaproteobacterium in the Family Ectothiorhodospiraceae, Isolated from a Brackish Spring in Death Valley, California. *Microbiol. Resour. Announc.* **9**, e01144-19 (2020).
35. Li, J. *et al.* Single-Cell Resolution of Uncultured Magnetotactic Bacteria via Fluorescence-Coupled Electron Microscopy. *Appl. Environ. Microbiol.* **83**, e00409-17 (2017).
36. Trubitsyn, D. *et al.* Complete Genome Sequence of Strain SS-5, a Magnetotactic Gammaproteobacterium Isolated from the Salton Sea, a Shallow, Saline, Endorheic Rift

Lake Located on the San Andreas Fault in California. *Microbiol. Resour. Announc.* **10**, e00928-20 (2021).

37. Lefèvre, C. T., Bernadac, A., Yu-Zhang, K., Pradel, N. & Wu, L.-F. Isolation and characterization of a magnetotactic bacterial culture from the Mediterranean Sea. *Environ. Microbiol.* **11**, 1646–1657 (2009).
38. Ji, B. *et al.* The chimeric nature of the genomes of marine magnetotactic coccoid-ovoid bacteria defines a novel group of Proteobacteria. *Environ. Microbiol.* **19**, 1103–1119 (2017).
39. Meldrum, F. C., Mann, S., Heywood, B. R., Frankel, R. B. & Bazylinski, D. A. Electron microscopy study of magnetosomes in a cultured coccoid magnetotactic bacterium. *Proc. R. Soc. Lond. B Biol. Sci.* **251**, 231–236 (1993).
40. Schübbe, S. *et al.* Complete Genome Sequence of the Chemolithoautotrophic Marine Magnetotactic Coccus Strain MC-1. *Appl. Environ. Microbiol.* **75**, 4835–4852 (2009).
41. Morillo, V. *et al.* Isolation, cultivation and genomic analysis of magnetosome biomineralization genes of a new genus of South-seeking magnetotactic cocci within the Alphaproteobacteria. *Front. Microbiol.* **5**, (2014).
42. Koziaeva, V. *et al.* Genome-Based Metabolic Reconstruction of a Novel Uncultivated Freshwater Magnetotactic coccus “Ca. Magnetaquicoccus inordinatus” UR-1, and Proposal of a Candidate Family “Ca. Magnetaquicoccaceae”. *Front. Microbiol.* **10**, (2019).
43. Abreu, F. *et al.* Cell Adhesion, Multicellular Morphology, and Magnetosome Distribution in the Multicellular Magnetotactic Prokaryote Candidatus Magnetoglobus multicellularis. *Microsc. Microanal.* **19**, 535–543 (2013).

44. Abreu, F. *et al.* 'Candidatus Magnetoglobus multicellularis', a multicellular, magnetotactic prokaryote from a hypersaline environment. *Int. J. Syst. Evol. Microbiol.* **57**, 1318–1322 (2007).

45. Abreu, F. *et al.* Deciphering unusual uncultured magnetotactic multicellular prokaryotes through genomics. *ISME J.* **8**, 1055–1068 (2014).

46. Leão, P. *et al.* Ultrastructure of ellipsoidal magnetotactic multicellular prokaryotes depicts their complex assemblage and cellular polarity in the context of magnetotaxis. *Environ. Microbiol.* **19**, 2151–2163 (2017).

47. Zhang, R. *et al.* Characterization and phylogenetic identification of a species of spherical multicellular magnetotactic prokaryotes that produces both magnetite and greigite crystals. *Res. Microbiol.* **165**, 481–489 (2014).

48. Kolinko, S., Richter, M., Glöckner, F.-O., Brachmann, A. & Schüler, D. Single-cell genomics reveals potential for magnetite and greigite biomineralization in an uncultivated multicellular magnetotactic prokaryote. *Environ. Microbiol. Rep.* **6**, 524–531 (2014).

49. Lefèvre, C. T. *et al.* A Cultured Greigite-Producing Magnetotactic Bacterium in a Novel Group of Sulfate-Reducing Bacteria. *Science* **334**, 1720–1723 (2011).

50. Lefèvre, C. T. *et al.* Comparative genomic analysis of magnetotactic bacteria from the Deltaproteobacteria provides new insights into magnetite and greigite magnetosome genes required for magnetotaxis. *Environ. Microbiol.* **15**, 2712–2735 (2013).

51. Sakaguchi, T., Burgess, J. G. & Matsunaga, T. Magnetite formation by a sulphate-reducing bacterium. *Nature* **365**, 47–49 (1993).

52. Sakaguchi, T., Arakaki, A. & Matsunaga, T. Desulfovibrio magneticus sp. nov., a novel sulfate-reducing bacterium that produces intracellular single-domain-sized magnetite particles. *Int. J. Syst. Evol. Microbiol.* **52**, 215–221 (2002).
53. Nakazawa, H. *et al.* Whole genome sequence of *Desulfovibrio magneticus* strain RS-1 revealed common gene clusters in magnetotactic bacteria. *Genome Res.* **19**, 1801–1808 (2009).
54. Shimoshige, H. *et al.* Isolation and cultivation of a novel sulfate-reducing magnetotactic bacterium belonging to the genus Desulfovibrio. *PLOS ONE* **16**, e0248313 (2021).
55. Lefèvre, C. T., Frankel, R. B., Pósfai, M., Prozorov, T. & Bazylinski, D. A. Isolation of obligately alkaliphilic magnetotactic bacteria from extremely alkaline environments. *Environ. Microbiol.* **13**, 2342–2350 (2011).
56. Li, J. *et al.* Intracellular silicification by early-branching magnetotactic bacteria. *Sci. Adv.* **8**, eabn6045 (2022).
57. Li, J. *et al.* Crystal growth of bullet-shaped magnetite in magnetotactic bacteria of the *Nitrospirae* phylum. *J. R. Soc. Interface* **12**, 20141288 (2015).
58. Lin, W. *et al.* Genomic insights into the uncultured genus 'Candidatus Magnetobacterium' in the phylum Nitrospirae. *ISME J.* **8**, 2463–2477 (2014).
59. Spring, S. *et al.* Dominating Role of an Unusual Magnetotactic Bacterium in the Microaerobic Zone of a Freshwater Sediment. *Appl. Environ. Microbiol.* **59**, 2397–2403 (1993).
60. Kolinko, S., Richter, M., Glöckner, F.-O., Brachmann, A. & Schüler, D. Single-cell genomics of uncultivated deep-branching magnetotactic bacteria reveals a conserved set of magnetosome genes. *Environ. Microbiol.* **18**, 21–37 (2016).

61. Uzun, M. *et al.* Detection of interphylum transfers of the magnetosome gene cluster in magnetotactic bacteria. *Front. Microbiol.* **13**, 945734 (2022).

62. Zhao, Y. *et al.* Insight into the metabolic potential and ecological function of a novel Magnetotactic Nitrospirota in coral reef habitat. *Front. Microbiol.* **14**, 1182330 (2023).

63. Kolinko, S. *et al.* Single-cell analysis reveals a novel uncultivated magnetotactic bacterium within the candidate division OP3. *Environ. Microbiol.* **14**, 1709–1721 (2012).

64. Rahn-Lee, L. *et al.* A Genetic Strategy for Probing the Functional Diversity of Magnetosome Formation. *PLoS Genet.* **11**, e1004811 (2015).

65. Grant, C. R., Rahn-lee, L. & Legault, K. N. Genome Editing Method for the Anaerobic Magnetotactic. *Appl. Environ. Microbiol.* **84**, 1–12 (2018).
