# Supplementary material for: A network of coiled-coil and actin-like proteins controls the cellular organization of magnetosome organelles in deep-branching magnetotactic bacteria
Source: Nat Commun. 2025 Dec 11;16:11453. doi: 10.1038/s41467-025-66326-2 (PMC12748827; doi:10.1038/s41467-025-66326-2)
Supplement: Supplementary file 3 — Reporting Summary [file 41467_2025_66326_MOESM3_ESM.pdf]

nature portfolio

Corresponding author(s): Arash Komeili
Last updated by author(s): 08/28/2025

Reporting Summary

Nature Portfolio wishes to improve the reproducibility of the work that we publish. This form provides structure for consistency and transparency in reporting. For further information on Nature Portfolio policies, see our Editorial Policies and the Editorial Policy Checklist.

Statistics

For all statistical analyses, confirm that the following items are present in the figure legend, table legend, main text, or Methods section.

n/a	Confirmed	
[ ]	[x]	The exact sample size (*n*) for each experimental group/condition, given as a discrete number and unit of measurement
[ ]	[x]	A statement on whether measurements were taken from distinct samples or whether the same sample was measured repeatedly
[ ]	[x]	The statistical test(s) used AND whether they are one- or two-sided<br>*Only common tests should be described solely by name; describe more complex techniques in the Methods section.*
[x]	[ ]	A description of all covariates tested
[ ]	[x]	A description of any assumptions or corrections, such as tests of normality and adjustment for multiple comparisons
[ ]	[x]	A full description of the statistical parameters including central tendency (e.g. means) or other basic estimates (e.g. regression coefficient) AND variation (e.g. standard deviation) or associated estimates of uncertainty (e.g. confidence intervals)
[ ]	[x]	For null hypothesis testing, the test statistic (e.g. *F*, *t*, *r*) with confidence intervals, effect sizes, degrees of freedom and *P* value noted<br>*Give P values as exact values whenever suitable.*
[x]	[ ]	For Bayesian analysis, information on the choice of priors and Markov chain Monte Carlo settings
[x]	[ ]	For hierarchical and complex designs, identification of the appropriate level for tests and full reporting of outcomes
[x]	[ ]	Estimates of effect sizes (e.g. Cohen's *d*, Pearson's *r*), indicating how they were calculated

Our web collection on statistics for biologists contains articles on many of the points above.

Software and code

Policy information about availability of computer code

Data collection	FEI Tecnai 12 TEM equipped with a 2k × 2k charge-coupled device camera (The Model 994 UltraScan®1000XP) at an accelerating voltage of 120 kV with Gatan Digtal Micrograph; IMG-MER; Acquity M-class ultra-performance liquid chromatography (UPLC) system that was connected in line with a Synapt G2-Si mass spectrometer; Spectronic 20D+ Spectrophotometer; Canon R50 camera with a RF35mm F1.8 Macro STM Lens
Data analysis	Geneious version 2025.2.1; ImageJ 1.54g; Microsoft Excel v 16.95.4; GraphPad PRISM (version 9 and version 10); FigTree v1.4.4; ChimeraX-1.09; MAFFT v 7.490; PhyMLTree v 3.3.20180621

For manuscripts utilizing custom algorithms or software that are central to the research but not yet described in published literature, software must be made available to editors and reviewers. We strongly encourage code deposition in a community repository (e.g. GitHub). See the Nature Portfolio guidelines for submitting code & software for further information.

Data

Policy information about availability of data

All manuscripts must include a data availability statement. This statement should provide the following information, where applicable:

- Accession codes, unique identifiers, or web links for publicly available datasets
- A description of any restrictions on data availability
- For clinical datasets or third party data, please ensure that the statement adheres to our policy

All data are available within the article and supplementary files, or available from the corresponding author upon reasonable request.

Research involving human participants, their data, or biological material

Policy information about studies with human participants or human data. See also policy information about sex, gender (identity/presentation), and sexual orientation and race, ethnicity and racism.

Reporting on sex and gender	n/a
Reporting on race, ethnicity, or other socially relevant groupings	n/a
Population characteristics	n/a
Recruitment	n/a
Ethics oversight	n/a

Note that full information on the approval of the study protocol must also be provided in the manuscript.

Field-specific reporting

Please select the one below that is the best fit for your research. If you are not sure, read the appropriate sections before making your selection.

[x] Life sciences [ ] Behavioural & social sciences [ ] Ecological, evolutionary & environmental sciences

For a reference copy of the document with all sections, see nature.com/documents/nr-reporting-summary-flat.pdf

Life sciences study design

All studies must disclose on these points even when the disclosure is negative.

Sample size	No statistical methods were used to predetermine sample size. We used numbers that are standard practice in the field by including at least three technical replicates. The information about number of replicates and sample sizes is provided in figure legends and supplementary information.
Data exclusions	No data were excluded from the analyses.
Replication	All TEM micrographs are representatives of the strains grown under the stated conditions. Each Cmag measurement experiment included three independent cultures. The growth assay and cellular fractionation experiments were repeated three or more times.
Randomization	Consistent with practices in the field, allocation of samples into experimental groups was not random. For some experiments, genetically identical strains were examined under varying growth conditions. In other experiments, the same condition was applied to strains that varied genetically in a known manner. Replicates were used to ensure consistency in the results. All particles that were capable of being measured were counted and measured in randomly selected cells.
Blinding	Consistent with practices in the field, investigators were not blinded. In many cases, blinding is not feasible since the effect of culture condition or mutation is apparent during data collection. Additionally, all experiments were replicated and were well-controlled through inclusion wild-type, mutant, and complemented strains.

Reporting for specific materials, systems and methods

We require information from authors about some types of materials, experimental systems and methods used in many studies. Here, indicate whether each material, system or method listed is relevant to your study. If you are not sure if a list item applies to your research, read the appropriate section before selecting a response.

Materials & experimental systems

n/a	Involved in the study	
[x]	[ ]	Antibodies
[x]	[ ]	Eukaryotic cell lines
[x]	[ ]	Palaeontology and archaeology
[x]	[ ]	Animals and other organisms
[x]	[ ]	Clinical data
[x]	[ ]	Dual use research of concern
[x]	[ ]	Plants

Methods

n/a	Involved in the study	
[x]	[ ]	ChIP-seq
[x]	[ ]	Flow cytometry
[x]	[ ]	MRI-based neuroimaging

Plants

Seed stocks	n/a
Novel plant genotypes	n/a
Authentication	n/a
